# Identification of a Lifespan Extending Mutation in the *Schizosaccharomyces pombe* Cyclin Gene *clg1* ^+^ by Direct Selection of Long-Lived Mutants

**DOI:** 10.1371/journal.pone.0069084

**Published:** 2013-07-09

**Authors:** Bo-Ruei Chen, Yanhui Li, Jessica R. Eisenstatt, Kurt W. Runge

**Affiliations:** 1 Department of Molecular Genetics, Cleveland Clinic Lerner College of Medicine at Case Western Reserve University, Cleveland, Ohio, USA; 2 Department of Genetics and Genome Sciences, Case Western Reserve University School of Medicine, Cleveland, Ohio, United States of America; 3 Department of Biochemistry, Case Western Reserve University School of Medicine, Cleveland, Ohio, United States of America; Newcastle University, United Kingdom

## Abstract

Model organisms such as budding yeast, worms and flies have proven instrumental in the discovery of genetic determinants of aging, and the fission yeast *Schizosaccharomyces pombe* is a promising new system for these studies. We devised an approach to directly select for long-lived *S. pombe* mutants from a random DNA insertion library. Each insertion mutation bears a unique sequence tag called a bar code that allows one to determine the proportion of an individual mutant in a culture containing thousands of different mutants. Aging these mutants in culture allowed identification of a long-lived mutant bearing an insertion mutation in the cyclin gene *clg1* ^*+*^. Clg1p, like Pas1p, physically associates with the cyclin-dependent kinase Pef1p. We identified a third Pef1p cyclin, Psl1p, and found that only loss of Clg1p or Pef1p extended lifespan. Genetic and co-immunoprecipitation results indicate that Pef1p controls lifespan through the downstream protein kinase Cek1p. While Pef1p is conserved as Pho85p in *Saccharomyces cerevisiae*, and as cdk5 in humans, genome-wide searches for lifespan regulators in *S. cerevisiae* have never identified Pho85p. Thus, the *S. pombe* system can be used to identify novel, evolutionarily conserved lifespan extending mutations, and our results suggest a potential role for mammalian cdk5 as a lifespan regulator.

## Introduction

The availability of model organisms, such as yeasts, worms and flies, with well-established, high-throughput genetics and lifespans shorter than those of mammals have greatly facilitated investigation of the evolutionarily conserved aspects of aging [1–3]. Genome-wide studies in model organisms are particularly powerful in the identification and characterization of longevity pathways. In *Caenorhabditis elegans*, systematic RNAi knockdown screens have revealed that reduced expression of mitochondrial genes and some genes required for viability, as well as genes involved in conserved processes such as insulin/IGF-1 and sirtuins can increase lifespan [4,5]. Work in the budding yeast *Saccharomyces cerevisiae* has also led to the discovery of conserved regulators of aging. Yeast aging can be monitored as both a chronological lifespan (CLS) and a replicative lifespan (RLS). CLS is the length of time during which a population of cells can maintain viability in a non-dividing state, also termed stationary phase [6]. In contrast, RLS is measured as the number of mitotic divisions that a given cell can undergo before senescence [7,8]. The budding yeast bar code-tagged ORF deletion mutant collection has greatly facilitated genome-wide studies [9,10], and large-scale screens on individual mutants from this collection have led to the identification of mutants defective in the TOR signaling and ribosomal biogenesis pathways with increased CLS and RLS [11,12]. The bar codes, unique DNA sequence tags associated with each mutation, have allowed investigators to screen for mutants with extended CLS in a pool of random mutants by determining the frequency of these bar codes, and thus the relative survival of each mutant, during the lifespan [13,14]. These high-throughput studies, referred to as “parallel analysis” of a mutant collection, provided evidence that genes involved in *de novo* purine biosynthesis, cell signaling, and fatty acid and tRNA metabolism also participate in cellular aging [13,14]. Our goal is to develop a combination of assays and approaches in *Schizosaccharomyces pombe* to use this evolutionarily distant and powerful genetic system to easily identify mutations in conserved pathways that extend lifespan.

The fission yeast *S. pombe* has recently emerged as a useful model for aging studies with unique advantages [15–19]. *S. pombe* diverged from budding yeast ~1 billion years ago and shares several processes with mammals that are absent in *S. cerevisiae* including the presence of an RNAi pathway, heterochromatic centromeres, splicing mechanisms and a requirement for the mitochondrial genome for survival [20–22]. Thus, *S. pombe* is a unique model system with the potential of discovering new longevity regulatory pathways while maintaining the same benefits as budding yeast such as facile modification of the genome and the ability to grow and analyze large populations.

We previously devised a CLS assay for *S. pombe* that both avoids a complication of the *S. cerevisiae* assay and allows for the direct selection of long-lived mutants. In *S. cerevisiae*, cell viability is analyzed as the percent of viable cells remaining after the culture has reached its maximal density, and followed until it has declined to 1% to 0.1% of its original value (e.g. from 10^8^ cfu/ml (CFU/ml) to 10^5^–10^6^ CFU/ml) [6,23]. This approach allows a simple comparison of yeast CLS assay results with lifespan assays of mammals, flies and worms, but has the major distinction in that a large number of individuals remain alive at the end of the study. In addition, measuring lifespan beyond the point when 0.1% of the remaining cells are viable is quite difficult because a subset of the yeast mutate and regrow as other cells die [24]. Because the ability to regrow may be distinct from slowed aging, one cannot easily select for long-lived survivors. However, clever screening techniques and the use of unique, genome-wide resources and bioinformatics have allowed the identification of mutants that increase longevity [12–14,25]. Our *S. pombe* CLS assay does not show this type of regrowth: cell survival gradually decreases until all of the cells in the culture have died. The assay recapitulates the evolutionarily conserved features of eukaryotic aging (e.g. extended CLS with caloric restriction, reduced CLS with over nutrition, lifespan regulation by Akt kinases) [16]. For microorganisms competing for survival in the wild, the ability of a small number of organisms to rapidly resume growth and consume nutrients as soon as they become available would confer a selective advantage. Our *S. pombe* CLS assay monitors the ability of cells to resume growth over a 10^5^ fold range in viability, and can thus utilize this aspect of microbial physiology to identify evolutionarily conserved pathways that regulate lifespan in all eukaryotes. In principle, one should be able to perform a direct selection for long-lived cells by simply aging a culture of mutants and identifying the long-lived mutants by their increased proportion in the surviving population. A challenge is distinguishing the long-lived mutants from those with normal lifespan that happen to survive until late in the assay.

One way to track the proportion of a mutant in a culture is to use mutants with bar code tags, as done in *S. cerevisiae* [13,14,26]. However, the *S. pombe* system does not have as many tools as *S. cerevisiae* for genome-wide and high-throughput studies (e.g. the bar-coded ORF deletion mutant collection and accompanying microarrays that can monitor bar code frequencies) [26]. We have generated an *S. pombe* DNA insertion mutant library in which each mutant bears a stable insertion tagged with a unique bar code [27]. A commercially available library of bar code-tagged *S. pombe* gene deletion mutants has also been constructed [28]. An advantage of our insertion mutant library is that it is designed to include viable haploid mutants in essential genes as well as mutations in non-coding RNAs, which are usually excluded from ORF deletion libraries [27]. Work in *C. elegans* has shown that reduced expression of genes required for survival can also affect aging [5], and our bar code-tagged insertion mutant library can test whether this property is evolutionarily conserved. A second advantage of our insertion library is that it was designed to allow screening for mutants that survive a treatment (e.g. aging) without prior knowledge of the bar code sequences and without a large investment in the production of microarrays. In contrast, these bar codes can be analyzed using standard cloning and sequencing approaches to identify those mutants with a growth advantage under a given condition [27].

In a proof-of-principle experiment, a portion of our insertion library was used to perform a screen for mutants with longer lifespans, and has identified a novel lifespan-extending mutation. A culture of several thousand mutant strains was aged, and mutants with longer lifespans were identified by the increased frequency of their associated bar codes in the surviving population. We identified a mutation in the fission yeast gene *clg1* ^*+*^, which encodes a protein with sequence similarity to budding yeast Clg1p, one of 10 cyclins that associate with the cyclin-dependent kinase (Cdk) Pho85p. We demonstrate that *S. pombe* Clg1p is one of three fission yeast cyclins that physically associates with the Cdk Pef1p, that only mutation of Clg1p or Pef1p extends lifespan, that Pef1p interacts with the kinase Cek1p and that lifespan extension by loss of Clg1p requires Cek1p. Surprisingly, four independent genome-wide screens in *S. cerevisiae* did not identify the Pef1p ortholog Pho85p as a lifespan regulator [11–14], showing that our *S. pombe* system can make novel contributions to the biology of aging. As the Pef1p family of Cdks is functionally related to human cdk5 [29,30], our results implicate a role for mammalian cdk5 and its cyclins in the chronological lifespan of human cells and show how the *S. pombe* system can be used to identify new evolutionarily conserved lifespan regulating pathways.

## Results

### Isolation of long-lived bar code-tagged insertion mutants using a novel bar code sequencing strategy

We developed a strategy that would allow identification of long-lived mutants from a pool of random mutants with wild type lifespans. Some long-lived mutants may have a longer median lifespan but the same maximum lifespan as wild type cells, while others may have both a longer median lifespan and a longer maximum lifespan (Figure S1A). As both types of mutants provide important information regarding the biology of aging, it is important to sample the population of aging cells at a point when both types of mutants are still alive and can be detected. Consequently, the population of random mutants needs to be sampled before all of the cells with a wild type lifespan have died (Figure S1B, gray bar) and the surviving cells will be composed of mutants with a wild type lifespan and those with extended lifespans. However, the long-lived mutants are expected to compose a larger proportion of the surviving cells compared to their proportion at the start of the experiment (Figure S1B). By using a mutant library where each mutation is tagged by a unique bar code, one can monitor the increased proportion of long-lived cells by monitoring the frequency of each bar code. The bar codes present at increased frequency can then be used to identify the original mutation.

**Figure 1:**
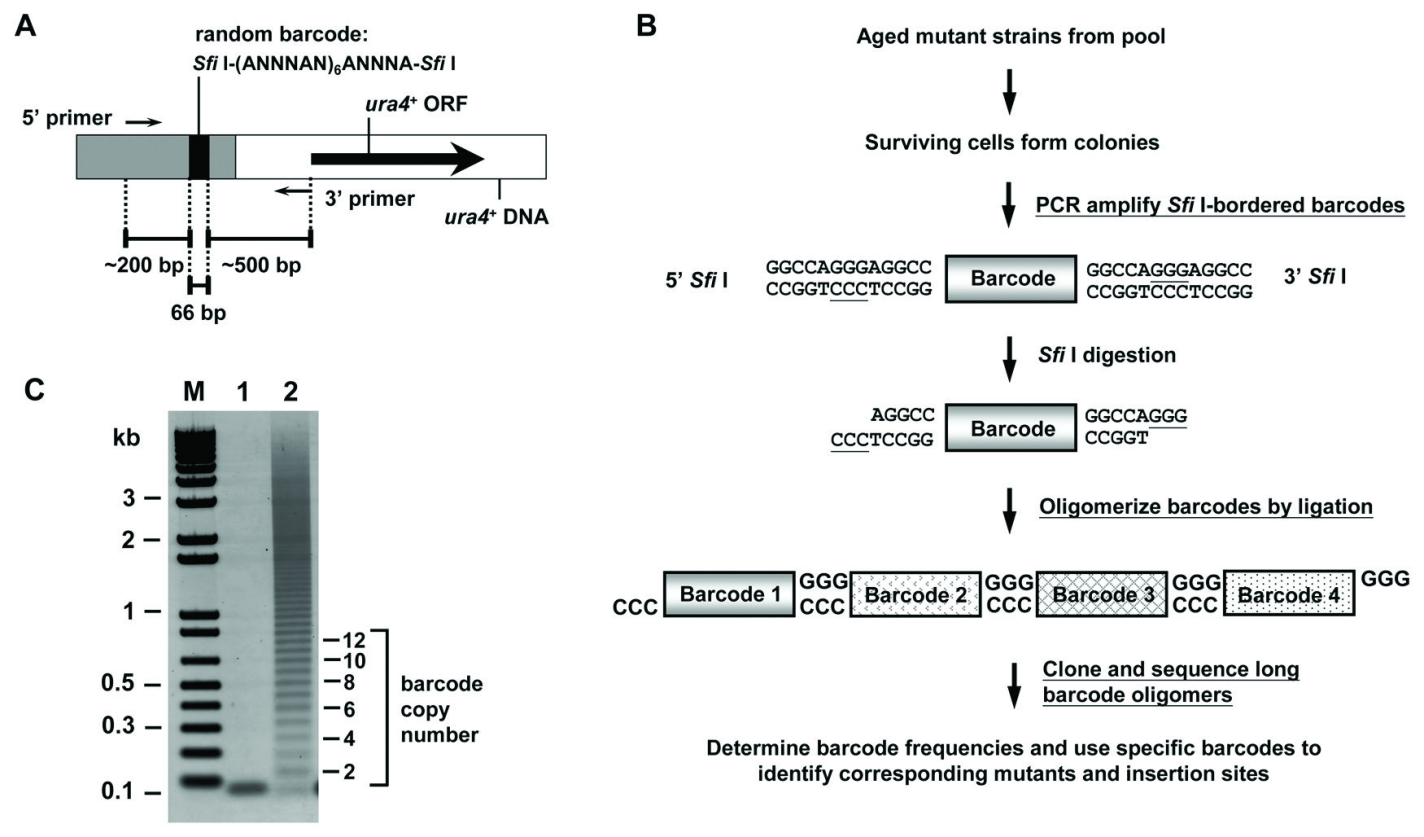
Isolating long-lived mutants using an *S. pombe* bar code-tagged insertion mutant library. (A) The schematic diagram of the insertion vector DNA used to generate the bar code-tagged *S. pombe* insertion mutants. The gray boxes represent sequences that protect the bar code from degradation. The 5’ and 3’ primers are to amplify the bar code-containing DNA. (B) The flow chart of the selection procedure. Cells from ~3,600 bar code-tagged insertion mutants were pooled together and aged in the standard SD medium. Bar codes with flanking *Sfi*I recognition sequences were amplified by PCR from the surviving cells, digested with *Sfi*I and ligated to produce long bar code oligomers for cloning so that many bar codes can be sequenced in a single reaction. As the bar code was designed to exclude *Sfi*I sites, all bar codes can be recovered in this procedure. (C) A representative bar code oligomerization. Purified bar code DNA monomer is shown in lane 1. After ligation, the bar code DNA monomer is converted to a series of higher molecular-weight oligomers (lane 2).

To allow the isolation of long-lived mutants using this approach, we generated a collection of bar code-tagged insertion mutants where each mutant contains a stably integrated insertion vector bearing a random bar code in an unknown genomic location (Figure 1A) [27]. While this library has the advantage of insertions in ORFs, 5’ and 3’ gene regulatory regions and non-coding RNAs, a disadvantage is that the insertions are random and complex which precludes their identification by high-throughput sequencing [27]. This disadvantage was overcome by including a 27 nt random bar code in each insertion that could be used to track the proportions of individual mutants without predetermining the bar code sequences, and to provide a unique primer to identify the insertion site. The bar codes are bordered by two *Sfi*I restriction enzyme recognition sequences. *Sfi*I does not cut in the bar codes and produces fragments with overhangs that allow bar code oligomerization (Figure 1A and 1B). By cloning long bar code oligomers, multiple bar code sequences can be determined in a single sequencing reaction, reducing the number of reactions required to determine bar code frequencies. Thus, one can sample the population of mutants near the end of the lifespan, amplify, oligomerize and clone the bar codes of the viable cells and determine the final bar code frequencies using common molecular biological techniques available in most laboratories. The bar code sequences found at high frequency can then be used to identify the long-lived mutants in the surviving cell population for further analysis.

We applied this approach in a genetic screen for CLS-extending mutations with ~3,600 bar-coded insertion mutants using our established aging assay [16]. Briefly, cells were inoculated at a low density (5 x 10^4^ cells/ml) into a defined medium, allowed to grow to stationary phase (~48 hr) and then samples were plated for viability as the culture aged. Near the end of the lifespan when viability had dropped by several orders of magnitude, multiple samples were plated to produce large numbers of individual colonies. Colonies of 600 surviving mutant cells from the culture on day 14, which had ~800 colony forming units (CFUs) per ml (Figure S2), were collected for stock plates and subsequent analyses.

To determine the bar code sequences in the 600 surviving mutant cells, bar codes and their flanking vector sequences were amplified by PCR, digested with *Sfi*I enzyme, and ligated to produce bar code oligomers between 0.3 to 1 kb (equivalent to pentamers to 16-mers) (Figure 1C), which were subsequently cloned and sequenced. A total of 405 bar codes were sequenced, which identified 38 different sequences (Table 1). Of the many bar codes identified, six bar code sequences composed a class isolated at high frequency (12 to 95 times) that accounted for ~88% of the sequenced bar codes (Table 1). These results suggested that the fraction of mutants bearing these bar codes increased as the population of cells in the selected culture were dying, consistent with our hypothesis for this selection (Figure S1B).

**Table 1:** Sequenced bar codes from surviving mutants.

**Frequencies of bar codes**	**Different types of bar codes^a^**	**% of all mutants examined**	**Affected gene(s)**
1	22	5.4	ND^b^
2	5	2.5	ND
3	3	2.2	ND
4	2	2.0	ND
12^c^	1 (4031)^e^	3.0	*clg1*+ ORF
19^c^	1 (4033)^e^	4.7	*clg1*+ ORF
69	1 (4032)^e^	17.0	*SPNCRNA.142*
73^d^	1 (4035)^e^	18.0	*SPRRNA.47* ^*f*^
88^d^	1 (4034)^e^	21.7	*SPRRNA.47* ^*f*^
95^d^	1 (4030)^e^	23.4	*SPRRNA.47* ^*f*^

^a^ The total number of determined bar code sequences is 405.

^b^ ND = not determined

^c^ The *clg1* ^-^ insertion mutant contained two bar codes.

^d^ The *sprrna.47* ^-^ insertion mutant contained three bar codes.

^e^ Representative bar code names

^f^ Insertion was located in the 28S ribosomal RNA coding gene array. The name of a representative gene is shown.

To exclude the possibility that increased bar code frequencies might result in part from a biased bar code (mutant) representation at the beginning of the experiment, bar codes from the starting pools prior to selection were also sequenced. We detected no bar codes significantly over-represented in this population (Table S1). Moreover, the bar codes identified in the initial culture were all different from those in the aged cultures (data not shown), indicating that the increased frequencies of the six bar codes in Table 1 were not a result of biased representation of mutants in the initial pool.

Using these six bar codes as specific PCR primers, we found that two bar codes (4031 and 4033) always identified the same mutant colonies, suggesting that these two bar codes co-existed in the same mutant. Similarly, bar codes 4030, 4034, and 4035 were also found in the same mutant colonies, while the bar code 4032 was found in a third, distinct set of colonies. Therefore, the six classes of enriched bar codes identified three mutants that increased in proportion in a pool of chronologically aged random mutants (Table 1).

### The enriched mutants contain insertions in clg1^+^, a non-coding RNA gene or the ribosomal RNA gene array

**Figure 2:**
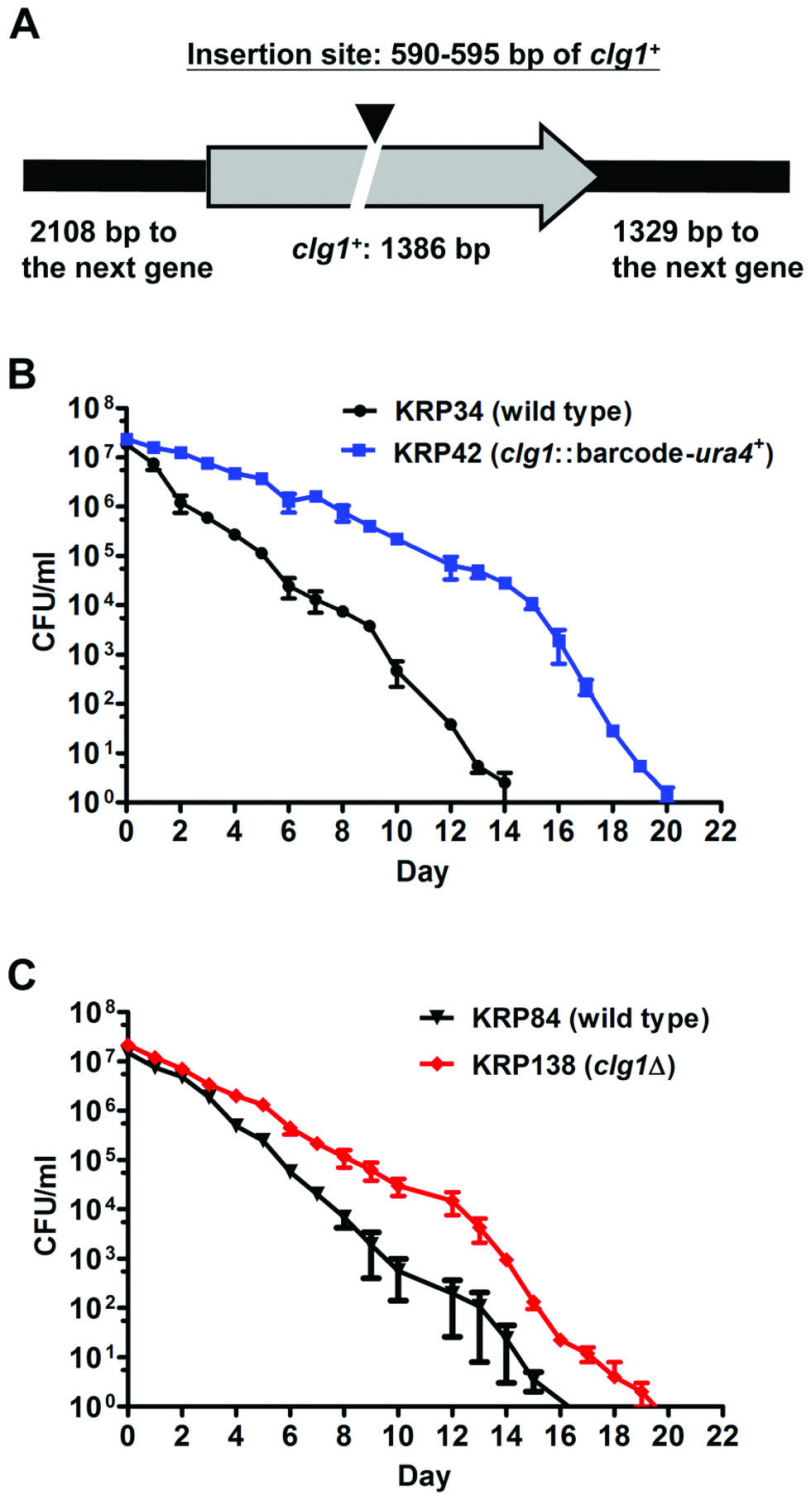
Insertion mutation or deletion of the *clg1* ^+^ ORF extends *S. pombe* CLS. (A) In the mutant carrying bar codes 4031 and 4033 (Table 1), tandem integration of two vectors (black triangle) was identified in the *clg1* ^+^ ORF and accompanied by a 4-bp deletion (Table 2). (B, C) The CLS of the re-generated mutant bearing the same *clg1* ^*-*^ insertion mutation in an isogenic wild type strain (KRP42, B), and a strain bearing a deletion of the *clg1* ^+^ ORF (KRP138, C) were determined in SD + 3% glucose medium (Materials and Methods). Both mutant strains exhibited a longer CLS than the wild type strain (p = 0.0001 for KRP42; p = 0.003 for KRP138). Error bars show the range of the duplicate cultures of each strain.

To identify the affected genes in these mutants, the insertions sites and types of insertions were identified using Thermal Asymmetric Interlaced-PCR (TAIL-PCR [31,32], Materials and Methods). A single insertion was in the coding portion of the *clg1* ^*+*^ gene accompanied by a 4 bp deletion (Figure 2A Table 2). This insertion consisted of two tandemly inserted vectors, accounting for the presence of two bar codes in this mutant (Table 1). A single insertion with no loss of genomic sequence was detected in the non-coding RNA gene *SPNCRNA.142* (Table 2). The insertion site in the third mutant identified by 3 bar codes (4030, 4034 and 4035, Table 1) could not be mapped by TAIL-PCR as the only sequences obtained were insertion vector sequences, indicating multiple integrations at the same site. By using the 4030 bar code sequence in a splinkerette PCR [27,33] approach, this insertion was mapped to the arrays of 28S ribosomal RNA genes on the ends of chromosome III (Table 2). Owing to the highly repetitive nature of the rRNA gene loci, the detailed structure of this insertion event was not pursued.

**Table 2:** Characterization of insertion events.

**Insertion site (chromosome)^a^**	**Insertion site (affected gene)^a^**	**Deletions (in bp) from the ends of the insertion vector^a^**		**Number of inserted vectors**
		**5’ end**	**3’ end**	
Chromosome 2; 1746832^a^-1746837	590-595^a^ bp of *clg1* ^*+*^ ORF (4 bp were deleted)	11 (1^st^ copy) 10 (2^nd^ copy)	5 (1^st^ copy) 976 (2^nd^ copy)	2
Chromosome 1; 214745^a^ − 214746	905^a^ − 906 bp of *SPNCRNA.142*	36	17	1
Chromosome 3	*SPRRNA.47* ^*b*^	ND^c^	ND^c^	≥ 3^d^

^a^ Insertion site (chromosome) shows the bases present at the 5’ and 3’ ends of the inserted DNA using the numbering from the *S. pombe* genome database (www.pombase.org) on May, 2011. Insertion site (affected gene) shows the bases that border the inserted DNA based on the number of bases from the A in the ATG of the ORF (for *clg1* ^+^) or the first nucleotide of the transcript (for *SPNCRNA.142*). The deletions from the ends of the insertion vector show the number of base pairs missing from the integrated insertion vector compared to the sequence of the DNA transformed into *S. pombe* cells. The ends of insertion sites were mapped by TAIL-PCR. The inserted vector ends were determined by PCR with flanking primers of the known insertion sites.

^b^ A representative gene of 100-150 copies of 28S rRNA coding genes.

^c^ ND = not determinable, due to the repetitive nature of rRNA arrays.

^d^ Based on the presence of three different bar codes in this mutant.

A secondary screen was performed to test the three most frequently identified mutants for increased lifespan. Colonies from the surviving cells were isolated and chronological lifespans were determined for wild type or mutant cells grown in individual cultures. The mutants bearing an insertion in either the *clg1* ^*+*^ gene or the 28S ribosomal RNA gene had longer lifespans, while the mutants bearing an insertion in the *SPNCRNA.142* non-coding RNA gene had a CLS very similar to wild type (Figure S3). Thus, two of the three mutants that appeared to have an extended lifespan in the mixed culture also had an extended lifespan in the individual cultures, and the *clg1* insertion mutation was analyzed further.

### Both the insertion mutation and complete deletion of the clg1^+^ ORF extend *S. pombe* CLS

To show that the long-lived phenotype was caused by the insertion mutation and not a secondary mutation that occurred during transformation, the portion of the *clg1* gene bearing the insertion was amplified from the original mutant (KRP44) and transformed into the original parental strain to create a new strain bearing the *clg1::*bar code-*ura4* ^*+*^ mutation. Two independent isolates of the re-generated *clg1* ^-^ insertion mutant (Figure 2B) showed extended CLS compared to the isogenic wild type strain. To determine whether this extended lifespan phenotype represented a loss of function mutation, a strain in which the entire *clg1* ^*+*^ ORF was deleted was generated (KRP138). Analysis of two independent transformants showed that the *clg1*Δ cells also lived longer than the wild type cells (Figure 2C). These results show that the longevity phenotype is due to the loss of *clg1* ^*+*^ function and indicate that the over-representation of the *clg1* ^-^ insertion mutant in the aged culture was a result of its increased CLS.

### Depletion of Clg1p and its associated cyclin-dependent kinase Pef1p extends *S. pombe* CLS

The Clg1p sequence encodes a cyclin domain (based on Pfam 25.0 database (http://pfam.sanger.ac.uk/)), and the loss of function *clg1* ^*-*^ insertion mutation separates the ATG from the predicted cyclin domain (Figure S4A). One of the potential *S. pombe* Clg1p homologs is *S. cerevisiae* Clg1p, which belongs to a cyclin family of 10 members that all associate with the Cdk Pho85p to regulate a variety of cellular processes [34,35]. We therefore searched the *S. pombe* proteome for a potential Pho85p ortholog and found that Pef1p had both the closest sequence similarity and had been previously identified as an *S. pombe* Cdk [36]. While little is known about the specific functions of the *S. cerevisiae* Clg1p/Pho85p complex, one Pho85p function related to CLS is that the Pho80p/Pho85p cyclin/Cdk complex negatively regulates the entry into quiescence through the protein kinase Rim15p [37–39]. As proper establishment of a quiescent state is important in the control of chronological aging [40,41], we hypothesized that, in fission yeast, Clg1p and Pef1p may regulate this process and the longevity phenotypes in the *clg1* ^*-*^ insertion and deletion mutants may be a consequence of defective Clg1p/Pef1p complex function.

**Figure 3:**
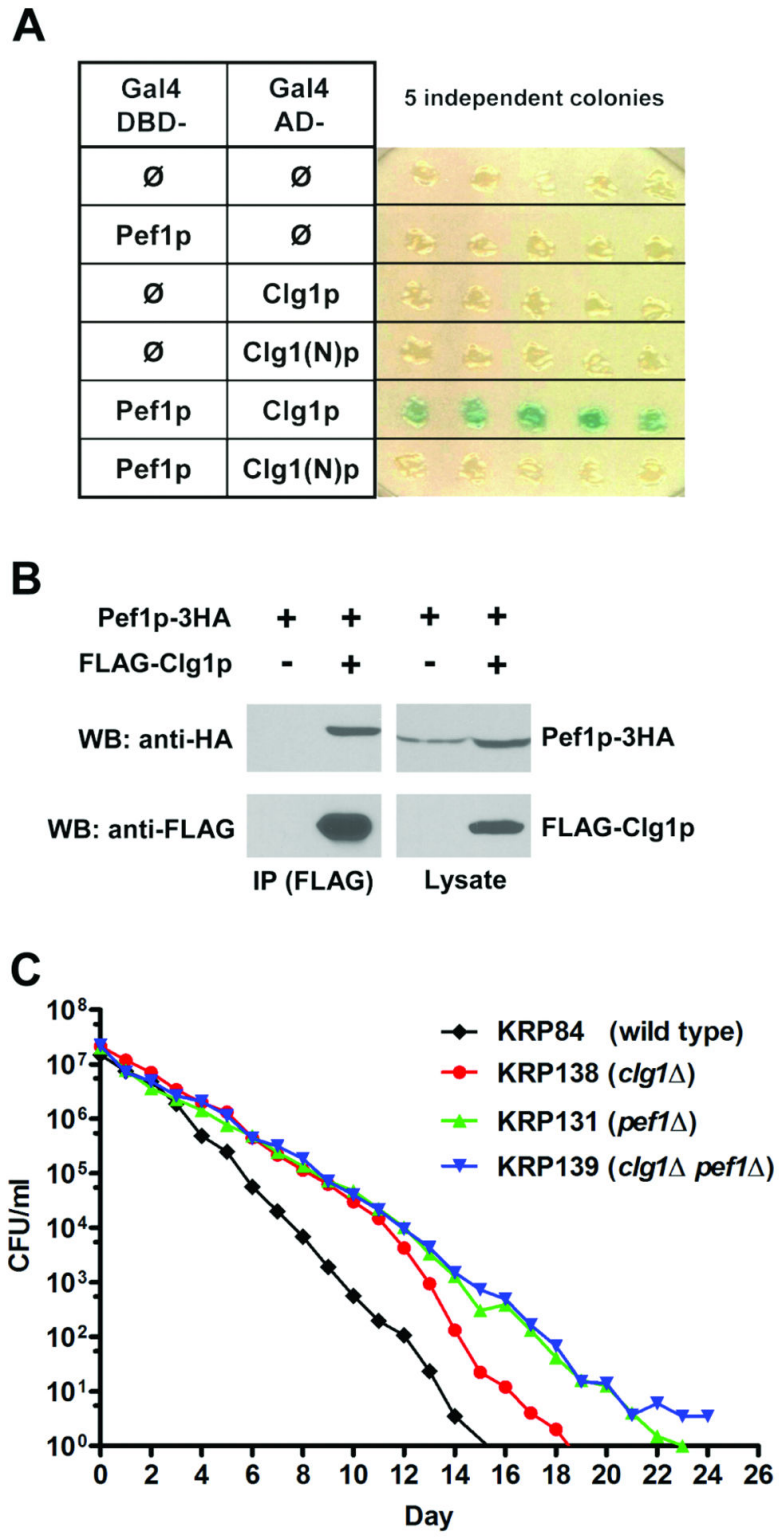
Clg1p and Pef1p interact and regulate CLS through the same pathway. (A) The two-hybrid assay of Clg1p and Pef1p. Cells expressing both Gal4 DNA binding domain (DBD)- and Gal4 activation domain (AD)-fusion proteins were analyzed as described in Materials and Methods. Blue color is indicative of a positive interaction whereas white color means no detectable protein interaction. Clg1(N) p is the N-terminal fragment predicted to be made in the *clg1* insertion mutation, and encodes the first 590 bps of the *clg1* ^+^ ORF. (B) Clg1p co-immunoprecipitates with Pef1p in *S. pombe* cells. Lysates prepared from cells expressing Pef1p-3HA and FLAG-Clg1p, or Pef1p-3HA alone, were immunoprecipitated with anti-FLAG antibody. The precipitated complexes and 100 µg of input lysate were analyzed by SDS-PAGE and Western blotting with anti-HA or anti-FLAG antibodies. (C) Deletion of *pef1* ^*+*^ extends CLS in the same pathway as *clg1* ^*+*^. The CLS of the *pef1*Δ single deletion mutant and the *clg1*Δ *pef1*Δ double deletion mutant were determined in SD + 3% glucose medium along side the wild type and *clg1*Δ mutant strain (Figure 2C). The lifespans of the two *pef1∆* mutants were longer than that of the wild type (p = 0.0045 for *pef1*Δ, p = 0.0029 for *clg1*Δ *pef1*Δ). The lifespan curve of the *clg1*Δ *pef1*Δ double deletion mutant was indistinguishable from the *pef1*Δ single deletion mutant, and the *clg1*Δ *pef1*Δ curve significantly overlapped with the *clg1*Δ mutant (p > 0.1 for all comparisons). Survival curves comparing each individual mutant with the wild type with error bars are shown in Figure S5.

Clg1p/Pef1p physical interaction was tested by yeast two-hybrid analysis and immunoprecipitation. In the two-hybrid analysis, the entire *pef1* ^*+*^ ORF was fused to the Gal4p DNA binding domain and the entire *clg1* ^*+*^ ORF was fused to the Gal4p activation domain, and evidence for a physical interaction was obtained (Figure 3A). In contrast, a fusion of the Clg1p N-terminal fragment predicted to be made in the insertion mutant, i.e. fusing the portion encoding amino acids 1-197 (Figure S4A) to the activation domain, showed no detectable interaction with Pef1p (Clg1(N) p, Figure 3A). Co-immunoprecipitation was also carried out to determine if this interaction occurs in fission yeast cells. FLAG-tagged Clg1p was expressed in cells that also expressed triple HA (3HA)-tagged Pef1p [36]. Western blotting of FLAG-Clg1p immunoprecipitates revealed the presence Pef1p-3HA (Figure 3B). A reciprocal co-immunocipitation experiment using anti-HA antibody also detected the association of Pef1p-3HA with FLAG-Clg1p (data not shown). These results indicate that Clg1p interacts with Pef1p in *S. pombe* cells and is a Pef1p-associated cyclin.

To determine if Clg1p and Pef1p are in the same genetic pathway that determines CLS, mutants lacking *clg1* ^+^, *pef1* ^+^ or both genes were generated and assayed for lifespan. Similar to the *clg1*Δ strain (KRP138) (Figure 2C), two independently constructed *pef1*Δ cells (KRP131) also exhibited a longer-than-wild type lifespan (Figure 3C). When both *clg1* ^+^ and *pef1* ^+^ were deleted, the lifespans of two independent double deletion mutants (KRP139) were not only longer than that of the wild type control, but also statistically indistinguishable from that of *clg1*Δ and *pef1*Δ (Figure 3C). The lifespan and physical interaction data support the hypothesis that these two proteins form a cyclin/Cdk complex to regulate chronological aging in *S. pombe*.

### Clg1p is the only known Pef1p-associated cyclin whose inactivation extends CLS

As budding yeast Pho85p kinase interacts with multiple cyclins [34,35], we determined whether *S. pombe* had additional Clg1p-like cyclins and whether these cyclins also regulate *S. pombe* chronological aging. The Pho85p-associated cyclins contain two domains: cyclin_N (PfamID: PF00134) and cyclin (Pfam ID: PF08613). These domains were used to search the *S. pombe* proteome for family members. The search using the cyclin_N domain identified 11 *S. pombe* proteins (Table S2). None of these 11 fission yeast sequences showed significant sequence similarity to the budding yeast Pho85p-associated cyclins and did not identify the previously known Pef1p-associated cyclin Pas1p. The search using the cyclin domain identified 3 *S. pombe* proteins: Clg1p, Pas1p and Spbc20f10.10p (Figure S4). All three of these proteins showed strong sequence similarity to Pho85p-associated cyclins: *S. pombe* Clg1p is similar to *S. cerevisiae* Clg1p, *S. pombe* Pas1p has strong sequence similarity to *S. cerevisiae* Pc15p and Spbc20f10.10p is most similar to *S. cerevisiae* Pcl7p.

**Figure 4:**
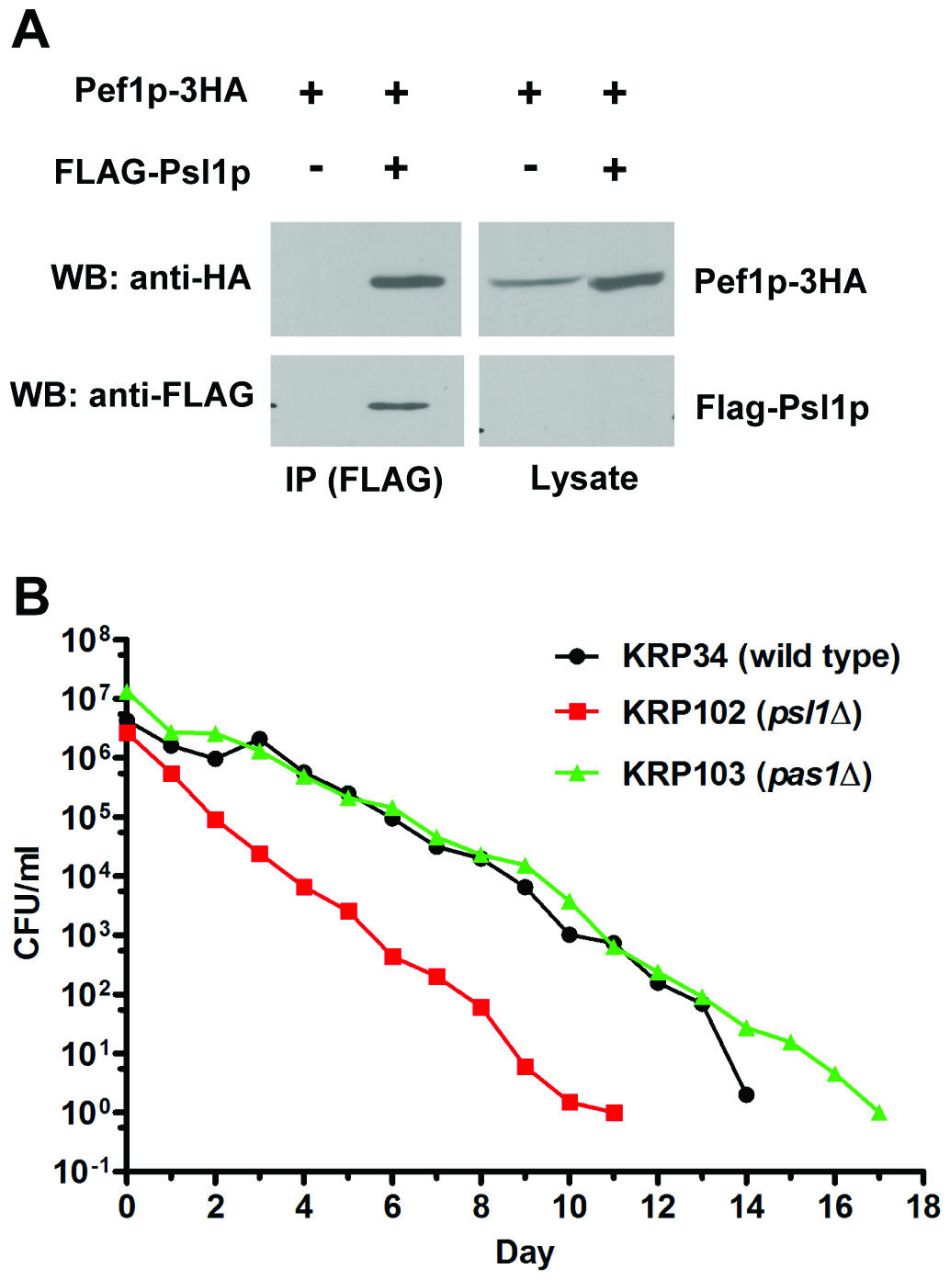
Deletion of the Pef1p-associated cyclins *pas1* ^+^ or *psl1* ^+^ does not extend *S. pombe* CLS. (A) Pef1p associates with the cyclin Psl1p. Cells expressing both Pef1p-3HA and FLAG-Psl1p (FLAG-Spbc20f10.10p) were lysed and immunoprecipitated with anti-FLAG antibody, followed by Western blotting with anti-FLAG and anti-HA antibodies. In the input lysate (100 µg), FLAG-Psl1p expression was too weak to be detected in the lysate. (B) Deletion of *pas1* ^*+*^ or *psl1* ^*+*^ does not extend *S. pombe* CLS. The *psl1∆* mutant had a shorter CLS than wild type cells (p= 0.0005) while the *pas1∆* mutant had a CLS indistinguishable from wild type cells (p>0.18). Survival curves comparing each individual mutant with the wild type with error bars are shown in Figure S6.

All three proteins were shown to interact with the *S. pombe* Pef1p kinase. Pas1p was previously shown to interact with the Pef1p kinase [36], similar to Clg1p (Figure 3). To test whether Spbc20f10.10p also interacts with Pef1p, FLAG-tagged Spbc20f10.10p was expressed in cells expressing Pef1p-3HA. Pef1p-3HA was detected in the anti-FLAG immunoprecipitates (Figure 4A), indicating that Spbc20f10.10p is also a Pef1p-associated cyclin. Based on its interaction with Pef1p and sequence similarity to budding yeast Pcl7p, the *SPBC20F10.10* gene has been given the common name *psl1* ^+^ for *P*cl *S*even *L*ike cyclin.

To address whether Pas1p and Psl1p may also regulate *S. pombe* chronological aging, the lifespans of strains lacking *pas1* ^+^ or *psl1* ^+^ were determined. In contrast to the *clg1* ^+^ ORF deletion, deletion of *psl1* ^+^ (*psl1*Δ, KRP102) shortened lifespan (Figure 4B). Deletion of *pas1* ^+^ showed no detectable effect on fission yeast chronological aging as the lifespan curve of the *pas1*Δ cells (KRP103) overlapped with that of the wild type control (Figure 4B). Therefore, CLS extension is a unique phenotype associated with loss of Clg1p.

### The increased CLS effected by loss of clg1^+^ requires the protein kinase Cek1p

In *S. cerevisiae*, the Pho80p/Pho85p complex phosphorylates the protein kinase Rim15p to control its subcellular localization and to antagonize Rim15p-dependent gene expression [37,42]. We therefore searched for and found two potential fission yeast orthologs with high sequence similarity to *S. cerevisiae* Rim15p: Cek1p and Ppk18p (Table S3). These two kinases were also similar to Rim15p in having split kinase domains interrupted between subdomain VII and VIII and a kinase activity regulating PAS domain (Table S3) [42–44].

**Figure 5:**
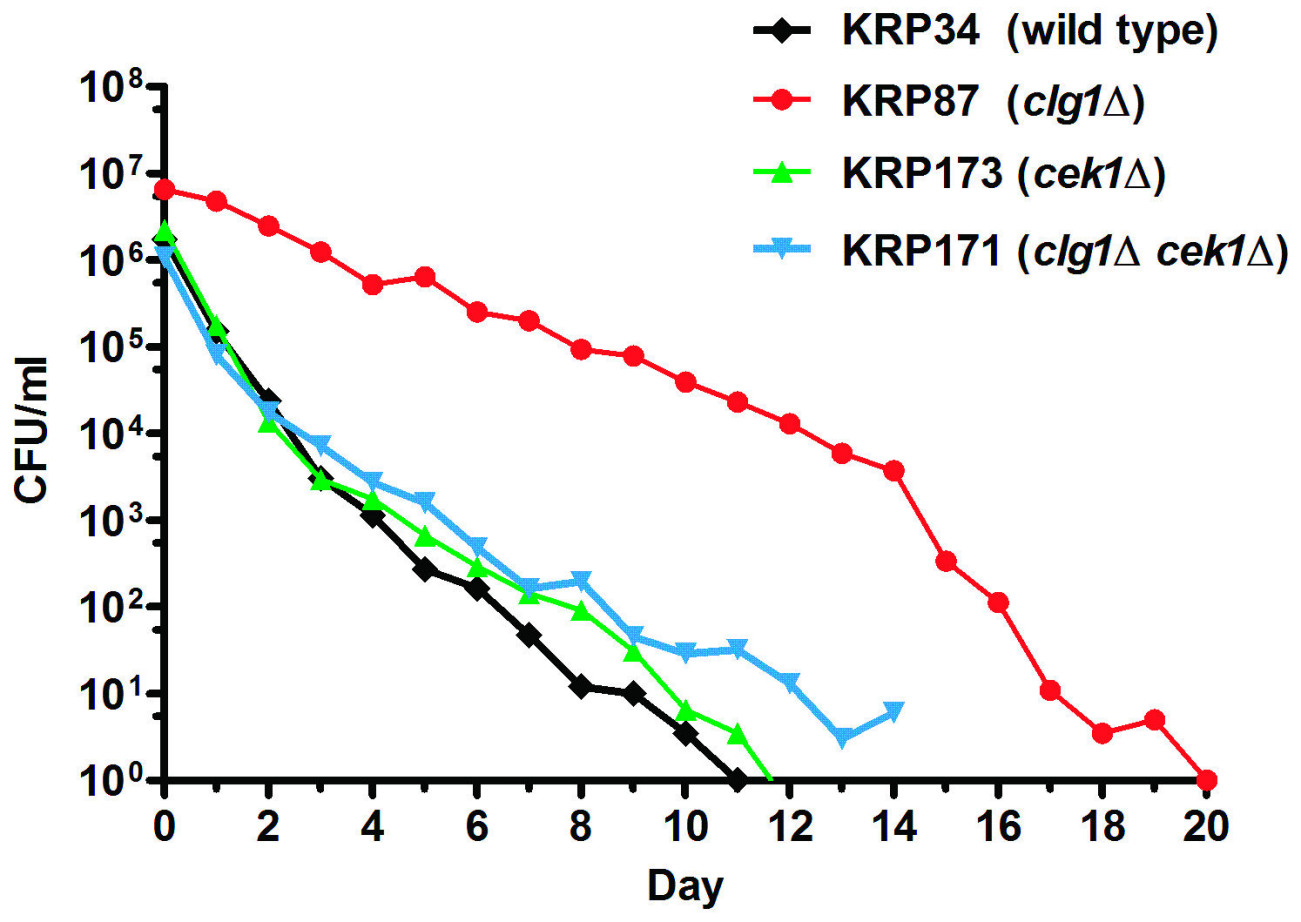
Deletion of *cek1* ^+^ abolishes the lifespan-extending effect of *clg1*Δ. While the *clg1*Δ mutant lifespan was longer than that of wild type cells (p = 0.0005), the *cek1*Δ mutant and the *clg1*Δ *cek1*Δ double mutant lifespans were not (p > 0.06 for *cek1*Δ, p > 0.6 for *clg1*Δ *cek1*Δ). The CLS of *cek1*Δ and *clg1*Δ *cek1*Δ were shorter than that of the *clg1*Δ mutant (p = 0.0002 for *cek1*Δ; p < 0.0001 for *clg1*Δ *cek1*Δ), but not significantly different from each other (p > 0.2). These strains reached the same density by day 0 the lifespan (~5 x 10^7^ cells/ml). Survival curves comparing each individual mutant with the wild type with error bars are shown in Figure S7.

If inactivation of Clg1p and Pef1p extends *S. pombe* CLS through the activity of Cek1p or Ppk18p, deletion of *cek1* ^+^ or *ppk18* ^+^ may block the lifespan extension phenotype caused by the *clg1*Δ mutation. Therefore, the lifespans of the *cek1*Δ and *ppk18*Δ single deletion mutants and the corresponding *clg1*Δ double deletion mutants were determined. Contrary to the long lifespan exhibited by the *clg1*∆ mutant, the *cek1*∆ single deletion mutant had a lifespan comparable to that of the wild type control (Figure 5). When *cek1* ^+^ was deleted in the *clg1*Δ mutant background, not only was the lifespan-extending phenotype of *clg1*Δ abolished, but the lifespan of the *cek1*Δ *clg1*Δ double-deletion mutant became very similar to that of the *cek1*Δ single mutant and the wild type strain (Figure 5). These data show that lifespan extension by *clg1* ^*+*^ deletion requires Cek1p, indicating that both proteins regulate chronological aging through an overlapping genetic pathway.

**Figure 6:**
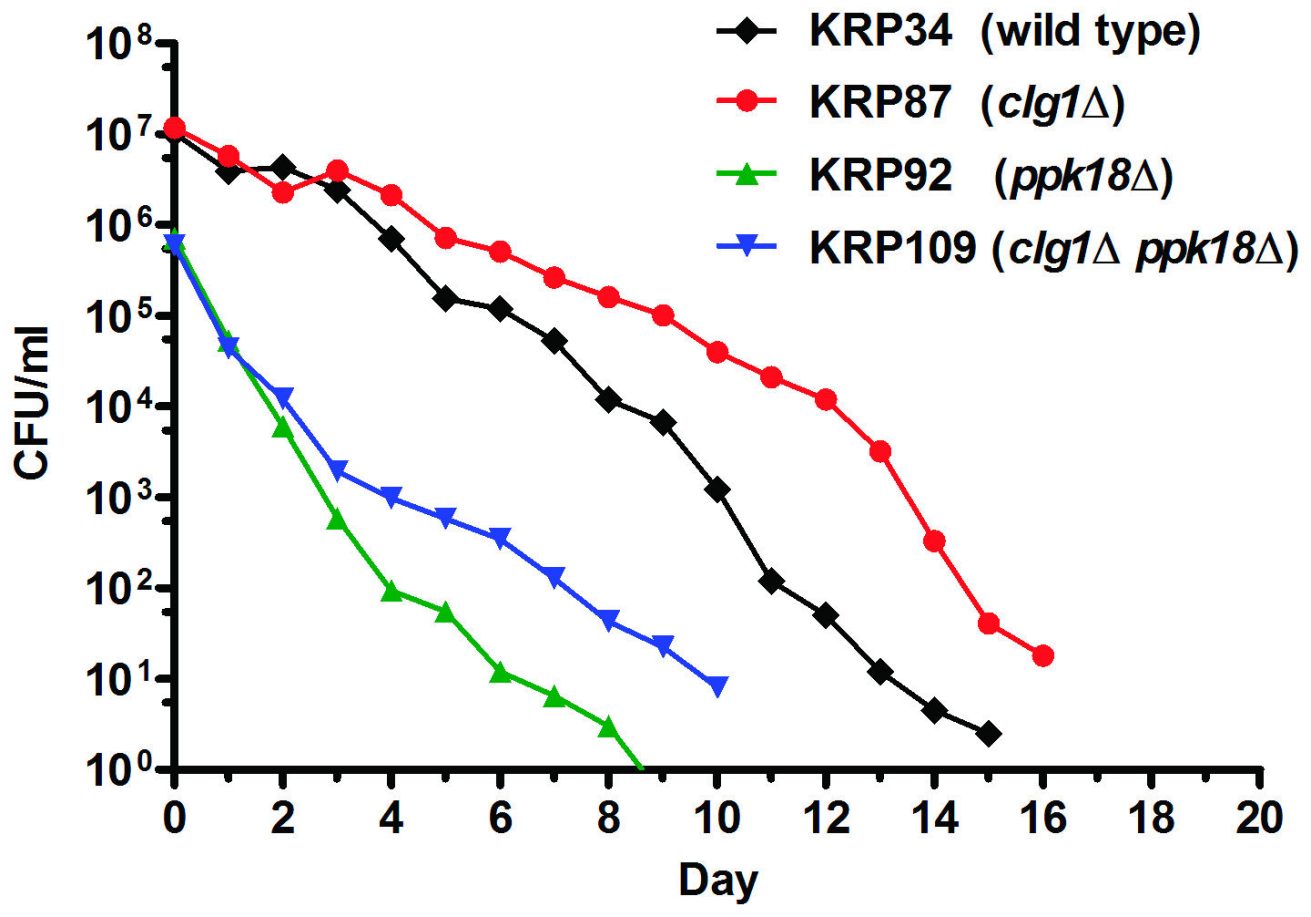
Deletion of *ppk18* ^+^ shortens CLS in both wild type and the *clg1*Δ strain. The CLS of the *ppk18*Δ and *clg1*Δ *ppk18*Δ mutants were shorter than that of the wild type (p = 0.002 for *ppk18*Δ; p = 0.001 for *clg1*Δ *ppk18*Δ). The *clg1*Δ *ppk18*Δ double deletion mutant had an intermediate lifespan, which is statistically different from that of *clg1*Δ (p = 0.001) and *ppk18*Δ (p = 0.0078 after Day 2). All four strains reached the same density by day 0 the lifespan (~5.7 x 10^7^ cells/ml). Survival curves comparing each individual mutant with the wild type with error bars are shown in Figure S8.

The *ppk18*Δ mutant behaved differently than the *cek1*Δ mutant in that deletion of *ppk18* ^+^ shortened lifespan compared to the wild type strain (Figure 6). Thus, Ppk18p is required for normal lifespan. The *ppk1∆ clg1∆* double deletion mutant also had a lifespan shorter than that of the wild type and *clg1∆* cells (Figure 6). The *ppk18∆ clg1∆* double deletion mutant consistently showed an intermediate length of lifespan that was between the short-lived *ppk18∆* and the long-lived *clg1* (Figure 6), suggesting that Ppk18p and Clg1p affect lifespan through non-overlapping genetic pathways.

**Figure 7:**
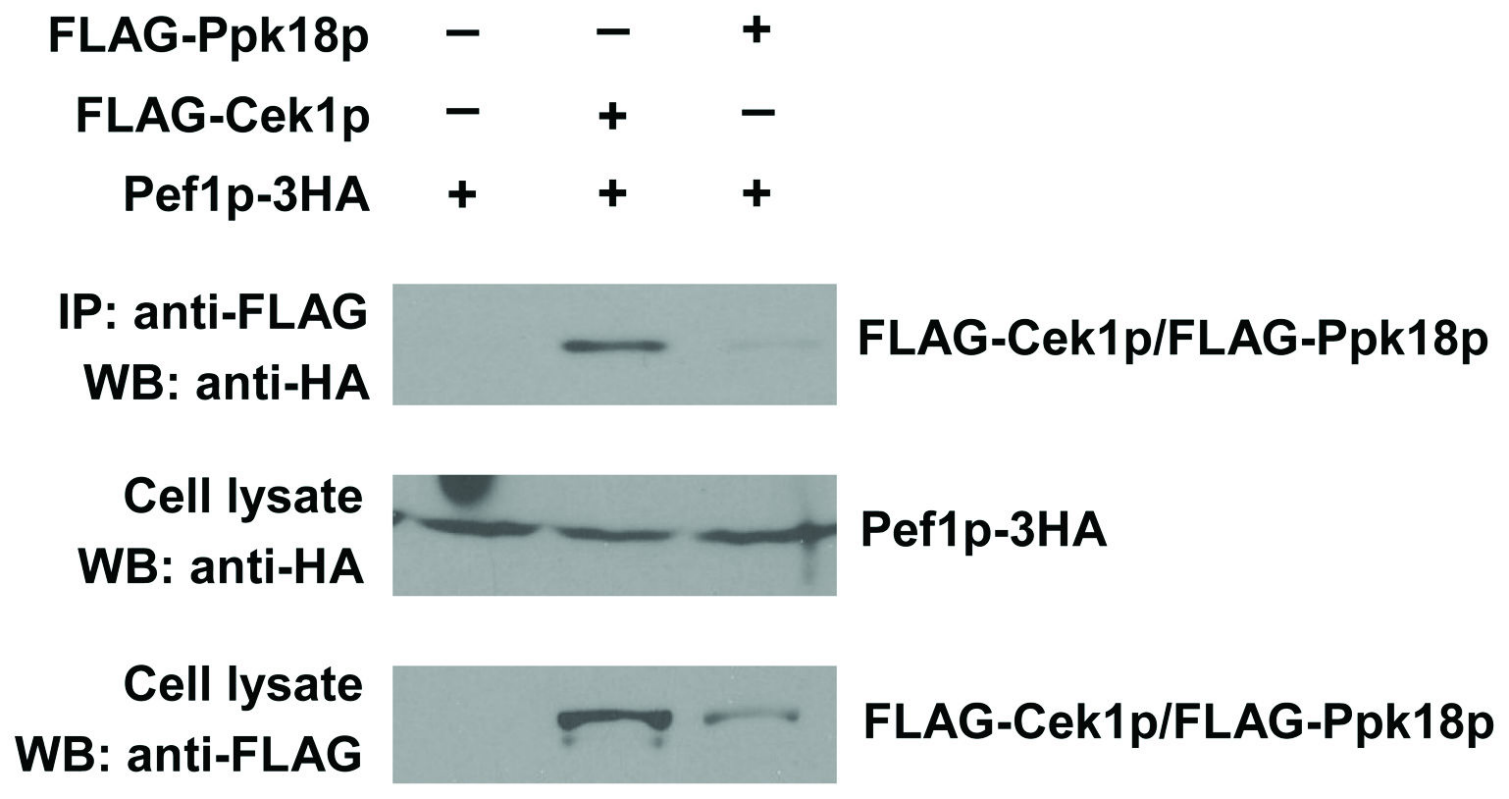
Pef1p interacts with Cek1p in fission yeast cells. Pef1p-3HA was expressed in cells with either FLAG-Cek1p or FLAG-Ppk18p. Cell lysates were subjected to immunoprecipitation with anti-FLAG antibody, and the immunoprecipitates and cell lysates were analyzed by Western blotting with anti-FLAG and anti-HA antibodies.

Pef1p physical interaction with Cek1p or Ppk18p was tested by co-immunoprecipitation. Pef1p-Cek1p association was clearly detected, but little or no interaction between Ppk18p and Pef1p was observed using the same assay (Figure 7). This result suggests that Pef1p acts directly on Cek1p, consistent with the hypothesis that Clg1p/Pef1p inhibits the action of Cek1p to extend CLS.

### Psl1p and Clg1p have antagonistic effects on the length of lifespan

The role of the novel Pef1p cyclin Psl1p in lifespan regulation was further characterized by examining cells that lack Psl1p and either Pef1p, Clg1p or Cek1p. Cells lacking both Psl1p and Pef1p had a long lifespan similar to cells lacking Pef1p (Figure S9A), indicating that the lifespan shortening caused by loss of Psl1p required its interacting Cdk. In contrast, cells lacking both Psl1p and Clg1p had a lifespan indistinguishable from wild type cells (*p* = 0.42, Table S5 Figure S9A) and different from either single mutant (Figures 2, 4). Thus, Psl1p and Clg1p have opposing roles in lifespan regulation.

The loss of both Psl1p and Cek1p had a small effect on lifespan compared to the two single mutants. The lifespans of cells lacking Psl1p or Cek1p were not significantly different from each other (Figure S9B, *p* = 0.08), and the lifespans of the stains lacking Cek1p or both Psl1p and Cek1p were also similar (*p* = 0.09, Table S5). In contrast, the lifespan of cells lacking Psl1p was slightly shorter than the double mutant lacking both Psl1p and Cek1p (*p* = 0.002, Table S5), indicating that cells required Cek1p for the complete lifespan shortening caused by loss of Psl1p. The sum of these double mutant tests indicate a role for Psl1p in lifespan regulation that counterbalances the effects of the Clg1p on the Pef1p pathway.

### The increased CLS effected by loss of clg1^+^ or pef1^+^ is not accompanied by increased stress resistance or by preventing medium acidification

Lifespan extension by caloric restriction and several gene mutations has been shown to strongly correlate with elevated stress resistance [25,45,46]. To test whether deletion of *clg1* ^+^ or *pef1* ^+^ also elicits increased stress resistance, we determined the sensitivity to hydrogen peroxide and a 55°C heat shock of wild type, *clg1*Δ, *pef1*Δ, *clg1*Δ *pef1*Δ and *cek1∆* cells grown to stationary phase (cells in day 1 cultures of a CLS assay). Wild type and all mutant cells were not sensitive to a low dose of H_2_O_2_ (150 mM), and *pef1*Δ, *clg1*Δ, *clg1*Δ *pef1*Δ and *cek1∆* cells showed no increased resistance to 450 mM H_2_O_2_ or to a 10 min exposure to 55°C (Figure S10). Therefore, lifespan extension by inactivation of Clg1p and Pef1p does not depend on enhanced stress resistance.

*S. cerevisiae* CLS can be limited by acidification of the culture medium, and some mutants that reduce medium acidification have a longer CLS [47,48]. To determine whether a similar process occurs in *S. pombe*, the pH of the culture medium for the first four days of a CLS assay was monitored for wild type, *clg1*Δ, *pef1*Δ, *clg1*Δ *pef1*Δ, *cek1∆* and *cek1∆ clg1∆* strains. At day 0 of the assay (two days after the culture was inoculated), the pH of the medium had dropped from 5.5 to 2.6 and remained a pH 2.6 for the rest of the assay (representative data are shown in Table 3). Thus, *S. pombe* cells do acidify the medium during the CLS assay, and the long-lived *clg1∆* and *pef1∆* mutants do not have extended lifespan because they fail to acidify the medium. Our observation is consistent with *S. pombe* results in rich medium which found no effect of pH changes on the length of CLS and no correlation between longevity and increased resistance to acetic acid [19].

**Table 3:** The pH and density of 96-hour cultures (day 2 of the CLS assay)^a^.

**Strains**	**pH**	**Density (cells/ml)**
SD + 3% glucose medium	5.47 (±0.005)	Not applicable
KRP84 (wild type)^b^	2.57 (±0.005)	5.15 x 10^7^ (±1.50 x 10^6^)
KRP131 (*pef1*Δ)	2.58 (±0.005)	3.38 x 10^7^ (±4.00 x 10^5^)
KRP138 (*clg1*Δ)	2.56 (±0.005)	5.61 x 10^7^ (±3.90 x 10^6^)
KRP139 (*pef1*Δ *clg1*Δ)	2.56 (±0.005)	5.05 x 10^7^ (±1.00 x 10^5^)
KRP34 (wild type)^b^	2.55 (±0.005)	4.70 x 10^7^ (±8.00 x 10^5^)
KRP173 (*cek1*Δ)	2.56 (±0.010)	4.66 x 10^7^ (±2.60 x 10^6^)
KRP171 (*cek1*Δ *clg1*Δ)	2.55 (±0.015)	4.94 x 10^7^ (±2.00 x 10^6^)

^a^ The pH and density of each strain are presented as the mean of cultures of two independent isolates with the ranges in parentheses.

^b^ The wild type KRP84 contains the same auxotrophic alleles as KRP131, KRP138 and KRP139; the wild type KRP34 has the same auxotrophic alleles as KRP173 and KRP171.

## Discussion

The fission yeast *S. pombe* is an emerging system in aging biology, with assays that recapitulate the features of aging that are conserved throughout eukaryotes [16,19]. Here we describe an approach that both greatly facilitates the use of the fission yeast model system by allowing the straightforward selection of long-lived strains and demonstrates how *S. pombe* can allow the discovery of novel lifespan-extending mutations. We coupled our previously validated *S. pombe* CLS assay with a random DNA insertion library designed to allow for the direct selection of long-lived mutants [16,27]. Even though the bar codes and insertion sites in the library are not known, our proof-of-principle experiment with a portion of the library identified a *clg1* ^*-*^ mutant with increased CLS. The *clg1* ^*-*^ mutant allowed us to then identify the interacting proteins Pef1p and Cek1p as regulators of *S. pombe* lifespan. As the Pef1p Cdk is conserved from yeast to humans and the human ortholog can complement fungal mutants lacking their Pef1p homolog [29,30], the lifespan regulating functions of the Clg1p/Pef1p pathway are likely to be conserved as well. An important aspect of this work is that four genome-wide screens in *S. cerevisiae* for mutants that extend CLS or RLS did not identify the Cdk Pho85p as a lifespan regulator [11–14]. Thus, the *S. pombe* system can make important, novel contributions to our understanding of longevity regulation.

The bar-coded insertion library approach has several advantages for developing new experimental model systems. Since prior knowledge of the bar code sequences and insertion sites is not required for a selection or screen, a large-scale investment in the construction of microarrays or high-throughput sequencing and bioinformatics is not necessary to identify important genes. A second major benefit is that the desired mutants can be identified even when a significant number of strains without the desired phenotype are still present. For example, aging is measured as a population-based phenomenon where some individuals die early in the lifespan and some die late. One consequence of this property is that in a mixed culture of many mutants, some cells from strains with wild type lifespans are expected to survive as long as cells from strains with increased lifespans (Figure S1). The results from the bar code analysis from our aging experiments were consistent with this prediction, as many different bar codes were isolated at low frequency (Table 1). In contrast, the bar codes for mutants bearing insertions in *clg1* ^*+*^ *, SPNCRNA.142* and *SPRRNA.47* were isolated at much higher frequency. It is important to note that the long-lived *clg1* ^*-*^ mutant that we identified was represented by less than 8% of the bar codes in the final population (Table 1) and would have been difficult to isolate by retesting individual survivors. This approach can therefore be applied to other screens where selected mutants are enriched but not free of non-mutant cells, such as mutations that partially increase the resistance to a drug or stress.

While we showed that our approach can directly select for long-lived mutants, an open question is whether all of the long-lived mutants in our proof-of-principle experiment were identified. The answer is unknown because the mutants present in the library are unknown. The complexity of these DNA insertions results in the absence of a defined sequence at the junction of the genomic DNA and insertion vector, and thus prevents the use of high-throughput methods to determine where the random insertions are in the genome [27]. Consequently, it is not known if long-lived mutants, such as *sck2* ^*-*^, *pka1* ^*-*^ [16,49] or *pef1* ^-^ (Figure 3) were present in the 3600 mutants screened, or what fraction of mutants contain insertions in or near ORFs expected to produce a long-lived phenotype. However, similar screens for long-lived mutants using the ~4800 viable *S. cerevisiae* ORF deletion mutants suggest that our selection in *S. pombe* yielded a similar fraction of mutants. Two groups identified non-overlapping sets of 12 [14] or 38 [13] long-lived mutants, giving yields of 0.25% or 0.79%, respectively, compared to our yield of 0.06%. Given that our random DNA insertion library would be expected to have a fraction of insertions with little or no phenotype while each ORF deletion strain has clearly lost one gene, the lower yield in our library is not surprising. In addition, the work in *S. cerevisiae* used microarrays to track the frequency of every bar code in the population, which may increase the sensitivity of detecting long-lived mutants compared to our analysis of 600 survivors. The future application of high-throughput sequencing to amplify all of the bar codes in our library and bioinformatics to track the relative proportion of each bar code in the experiment may help identify additional long-lived mutants in the insertion library.

The isolation of two different, non-overlapping sets of long-lived *S. cerevisiae* mutants [13,14] shows that differences in the aging assay can significantly alter which mutants are scored as long-lived. Thus, altering the conditions of the aging assay might identify additional genes that regulate lifespan under different conditions. For example, *S. pombe* has two Akt kinases encoded by the *sck1* ^*+*^ and *sck2* ^*+*^ genes, and loss of *sck1* ^*+*^ only increases lifespan under conditions of over nutrition, while loss of *sck2* ^*+*^ increases lifespan under normal and over nutrition conditions [16]. Thus, re-assaying both the *S. cerevisiae* and *S. pombe* libraries under altered conditions should reveal additional lifespan regulating genes.

An alternative approach to aging mixtures of mutants in a single culture has been to assay each *S. cerevisiae* ORF deletion mutant in a single microtiter well [12]. In this approach, mutants with a growth disadvantage in a mixed culture can still be assayed and the CLS changes monitored. This approach was used to rank the lifespans of almost all mutants of the ORF deletion set, and therefore could identify considerably more long-lived mutants than the mixed culture approaches. A major outcome of the microtiter plate approach was the identification of genes regulated by the TOR pathway as important to extending CLS [12], which were not identified in the mixed culture approaches [13,14]. While individual cultures of *tor* ^-^ pathway mutants do show extended lifespan, most of these strains grow more slowly than wild type and show reduced growth compared to most of the mutants in the *S. cerevisiae* ORF deletion set [50,51]. We have observed similar slow growth for the *S. pombe tor1*Δ mutant as well (B–RC and KWR, unpublished observations). This growth disadvantage may have prevented the identification of the Tor pathway in the mixed culture experiments. A second major discovery with the microtiter well assay was that acidification of the culture medium is major determinant of longevity as many of the long-lived strains appeared to reduce acidification, leading these researchers to suggest that alternative medium conditions may be more informative for modeling aging in metazoans [47,48]. This consideration underscores the potential of the *S. pombe* system, as the mutants we identified acidify the medium, still extend lifespan and identify a pathway conserved in metazoans [19] (Table 3).

**Figure 8:**
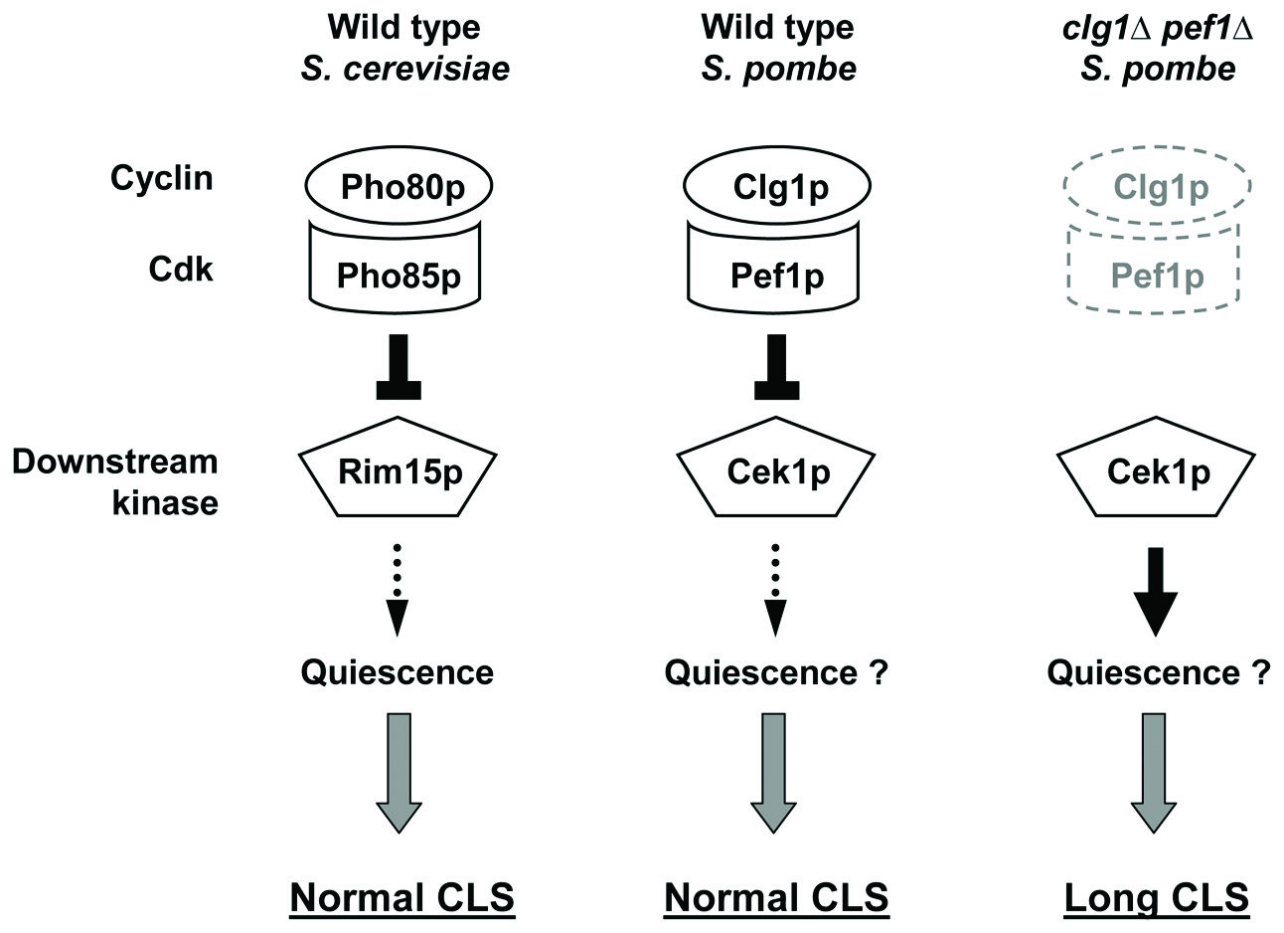
A hypothesis of Clg1p/Pef1p-dependent control of chronological aging through the Cek1p kinase. In budding yeast, Pho80p and Pho85p have been shown to negatively regulate Rim15p activity (left). Active Rim15p promotes entry into quiescence and is required for normal CLS [37]. Thus, Pho80p/Pho85p negatively regulate cell cycle exit. Identification of the homologous cyclin Clg1p, Cdk Pef1p and downstream kinase Cek1p in *S. pombe* indicates that these proteins may also modulate entry into quiescence (middle). As efficient entry into quiescent states is important for long chronological lifespan, we propose that inactivation of Clg1p and/or Pef1p removes the repression of the Cek1p effector kinase, and thus stimulates entry into quiescence (the thick black arrow on the right) and extends CLS.

The isolation of the long-lived *clg1* ^-^ insertion mutant led to our identification of the Pef1p Cdk and Cek1p PAS kinase as interacting proteins that control lifespan, and that Clg1p and Psl1p have antagonistic roles in lifespan regulation. These data are consistent with the hypothesis that Clg1p/Pef1p-Cek1p compose a signaling module similar to Pho80p/Pho85p-Rim15p [37,42]. These two pathways show different evolutionary adaptations in these two divergent yeasts. While loss of Pef1p extends lifespan (Figure 3), loss of Pho85p does not [11–14]. In addition, loss of budding yeast Rim15p reduces stress resistance [37,42], while loss of fission yeast Cek1p does not (Figure S10). Interestingly, the Pho80p/Pho85p-Rim15p pathway does control entry into quiescence [37,42], a crucial event for long CLS [40,41], which could potentially explain the extended lifespans of the *clg1∆, pef1∆*, *clg1∆ pef1∆* and *psl1∆ pef1∆* mutants (Figures 3, 5 and S9). Thus, one simple hypothesis to explain our data is that the Pho80p/Pho85p-Rim15p and Clg1p/Pef1p-Cek1p pathways affect the entry into quiescence such that loss of the cyclin/Cdk enhances survival in stationary phase (Figure 8), which will require additional tests in the future. This proposed function for Clg1p/Pef1p is distinct from the Pas1p/Pef1p complex, which has been implicated in the G1-S transition of the cell cycle [35], and suggests that Pef1p controls a diverse array of cellular processes similar to budding yeast Pho85p [34,35].

The identification of the Clg1p/Pef1p lifespan regulatory pathway integrates well with earlier work showing that loss of the *S. pombe* Akt kinases (Sck1p and Sck2p) and Protein kinase A (Pka1p or PKA) can extend CLS [16,49]. Mutation of the related genes in *S. cerevisiae*, the kinase Sch 9p and the PKA regulator Cyr1p, extends lifespan in pathways that require Rim15p [25,52]. The similarities between the Clg1p/Pef1p-Cek1p and Pho80p/Pho85p-Rim15p pathways suggest that the *S. pombe* Akt and PKA lifespan regulatory functions may also require Cek1p. Given the differences in the phenotypes of *S. cerevisiae* and *S. pombe* cells to loss of these signaling components, it will be interesting to determine whether other *S. cerevisiae* Rim15p-dependent pathways are conserved *S. pombe*.

The Clg1p/Pef1p-Cek1p module appears to be well-conserved in eukaryotes as homologs for each protein can be found in worms, fruit flies, and humans (Table S4) and that the human cdk5 can substitute for Pho85p in some yeast functions [30,53]. The activity of cdk5 is required for senescence in flies and mammals, and impairing cdk5 activity has been shown to cause neuronal cell death [54–56], a phenotype thought to be associated with quiescent neurons re-entering the cell cycle [57]. Thus, the *S. pombe* Pef1p pathway with its three known cyclins provides a tractable genetic model that may provide insight to the cellular lifespan of neuronal cells. The *S. pombe* proteins also reveal new potential overlapping functions with mammalian cells. Cek1p has been shown to have functions impacting mitosis and the DNA damage response [58,59]. The human homologs of Cek1p, MASTL and LATS (Table S4), have also been implicated in different aspects of mitosis [60–63]. As genomic instability is another process related to aging and aging-associated syndromes [64,65], the evolutionarily conserved Clg1p/Pef1p-Cek1p module may control several different processes that impact CLS. The relatively simple three cyclin-Pef1p system in *S. pombe* will be an ideal system for testing these ideas.

## Materials and Methods

### Strains and media

The fission yeast strains used in this study are listed in Table 4. Single ORF deletions were constructed by transforming wild type cells with fragments containing ~500 bp of genomic sequence immediately 5’ to the ORF, the selectable marker and ~500 bp of genomic sequence immediately 3’ to the ORF. Fragments were constructed by overlap PCR and sequenced to ensure that the genomic DNA exactly matched the published sequence. Double deletions strains were constructed by genetic crosses and tetrad dissection. The ~3600 mutants screened for CLS-extending mutations are from our bar code-tagged fission yeast insertion mutant library (pools 1 and 2, described in [27]).

**Table 4:** Fission yeast strains used in this study.

**Strain**	**Genotype**	**Comment**
KRP1	h^-^ *ade6-M216 ura4-D18 leu1-32 his7-366*	[16,70]
KRP34	h^-^ *ade6-M216 leu1-32 his7-366*	
KRP83	h^-^ *ade6-M216 ura4-D18 his7-366*	
KRP84	h^-^ *ade6-M216 his7-366*	
KRP42	h^-^ *ade6-M216 ura4-D18 leu1-32 his7-366 clg1*: :*bar code-ura4* ^*+ a*^	Re-constructed mutant
KRP44	h^-^ *ade6-M216 ura4-D18 leu1-32 his7-366 clg1*: :*bar code-ura4* ^*+ a*^	Bar-coded library mutant; original mutant isolate from the selected culture
KRP87	h^-^ *ade6-M216 ura4-D18 leu1-32 his7-366 clg1*Δ*::ura4* ^+^	
KRP88	h^+^ *ade6-M216 ura4-D18 leu1-32 his7-366 clg1*Δ*::ura4* ^+^	
KRP131	h^-^ *ade6-M216 ura4-D18 his7-366 pef1*Δ*::ura4* ^+^	
KRP138	h^-^ *ade6-M216 leu1-32 his7-366 clg1*Δ*::leu1* ^+^	
KRP139	h^-^ *ade6-M216 ura4-D18 leu1-32 his7-366 clg1*Δ*::leu1* ^+^ *pef1*Δ*::ura4* ^+^	
KRP92	h^-^ *ade6-M216 ura4-D18 leu1-32 his7-366 ppk18*Δ*::ura4* ^+^	
KRP109	h^-^ *ade6-M216 ura4-D18 leu1-32 his7-366 clg1*Δ*::ura4* ^+^ *ppk18*Δ*::ura4* ^+^	Derived from a cross of KRP88 and KRP92
KRP70 (BG4355H)	h^+^ *ade6-M210 ura4-D18 leu1-32 cek1*Δ*::KanMX*	Bioneer *S. pombe* haploid deletion set version1
KRP171	h^-^ *ade6-M210 ura4-D18 leu1-32 his7-366 clg1*Δ*::ura4* ^+^ *cek1*Δ*::KanMX*	Derived from a cross of KRP70 and KRP87
KRP173	h^-^ *ade6-M216 leu1-32 his7-366 cek1*Δ*::KanMX*	Derived from a cross of KRP70 and KRP34
KRP102	h^-^ *ade6-M216 ura4-D18 leu1-32 his7-366 psl1*Δ*::ura4* ^+^	
KRP103	h^-^ *ade6-M216 ura4-D18 leu1-32 his7-366 pas1*Δ*::ura4* ^+^	
K566-11	h^-^ *ura4-D18 pef1*::*pef1HA* ^+^-*ura4* ^+^ *leu1-32*	[36]
KRP130	h^-^ *ade6-M216 leu1* ^+^ *ura4-D18 his7-366 cek1*Δ*::ura4* ^+^	
KRP283	h^-^ *ade6-M216 leu1-32 ura4* ^+^ *his7-366 psl1*Δ*::leu1* ^+^	
KRP281	h^-^ *ade6-M216 leu1-32 ura4-D18 his7-366 psl1*Δ*::leu1* ^+^ *clg1*Δ*::ura4* ^+^	
KRP282	h^-^ *ade6-M216 leu1-32 ura4-D18 his7-366 psl1*Δ*::leu1* ^+^ *cek1*Δ*::ura4* ^+^	
KRP280	h^-^ *ade6-M216 leu1-32 ura4-D18 his7-366 psl1*Δ*::leu1* ^+^ *pef1*Δ*::ura4* ^+^	

^a^ These genes were disrupted by the insertion vectors containing both *ura4* ^*+*^ and the insertion vector DNA sequences as described elsewhere [27].

Fission yeast growth media used in this study were yeast extract + 225 mg/l of supplements + 3% glucose (YES) [66], synthetic dextrose + 150 mg/l each of the supplements adenine, uracil, leucine, histidine + 3% glucose (SD medium) [16,67] and Edinburgh minimal medium (EMM) + 225 mg/l each of the supplements adenine, uracil, leucine, histidine + 2% glucose [66]. Plates contained the same medium with addition of 2% agar.

### Chronological aging assays

Chronological aging assays were performed as described in Chen and Runge [16]. Briefly, cells were seeded at the initial cell density of 5 x 10^4^ cells/ml in 125-ml flasks containing 30 ml of SD medium + 3% glucose and maintained in an enclosed air platform shaker rotating at 220 rpm at 30°C. Cultures were grown for two days to reach maximum cell density, and this time point was designated as day 0. For the strains in this work, all final cell densities at day 0 were between 4 x 10^7^ to 7 x 10^7^ cells/ml. Aliquots of cultures were then taken on the days indicated in the Results, and multiple dilutions were plated on YES plates in duplicate and grown at 30°C for four days. The numbers of colonies formed on YES plates were used to calculate the number of colony forming units per ml (CFU/ml) of culture. For each experiment, CFUs were monitored until they reached < 10/ml. Each aging assay used two independent isolates of each gene deletion mutant to obtain the survival curve and range of values. In experiments comparing different mutants, all aging assays were performed at the same time. Statistical comparison of different chronological lifespan curves was performed using the Wilcoxon signed rank test in Prism 4 (GraphPad Software). A summary of the statistical comparisons are shown in Table S5.

### Isolation of long-lived mutants by ligation-mediated bar code sequencing

To screen for long-lived mutants, two independent freezer stocks each composed of ~1800 *S. pombe* bar code-tagged insertion mutants were thawed on ice, plated on complete EMM plates with supplements except uracil (~10^5^ cells/plate, 12 plates for each mutant pool) and grown at 30°C for five days. Revived cells were scraped off the plates and resuspended into sterile milliQ water to inoculate cultures at the density of 5 x 10^4^ cells/ml in 1000-ml flasks with 240 ml of SD medium + 3% glucose. The CFUs of the culture were monitored at 30°C with 220 rpm shaking for 15 days.

To identify the bar codes enriched in the surviving cells, a total of 600 colonies from the cells plated on day 14 were recovered. To facilitate the isolation of enriched mutants, these 600 colonies were divided to six groups of ~100 colonies each for genomic DNA preparation and subsequent analyses. DNA fragments (~750 bp) containing bar codes were amplified from genomic DNA of the surviving cells by PCR (using primers BarcodePCR(888r) and hsplam6, Figure 1A and Table S6). The amplified DNA was digested with *Sfi*I and separated on a 2% low melting agarose gel. The gel slice containing the 66 bp bar code DNA fragment was melted at 65°C with 100 µl of TE (10 mM Tris-HCl pH 8.0, 1 mM EDTA), 70 µl of 3M sodium acetate, pH 5.2 and then mixed with 0.6 ml of TE-saturated phenol by vortexing prior to centrifugation at 13,200 rpm for 5 minutes. The aqueous phase was re-extracted with 0.6 ml of phenol/chloroform/isoamyl alcohol (25:24:1; vol: vol: vol), followed by extraction with 0.6 ml of chloroform/isoamyl alcohol (24:1; vol: vol). The final aqueous phase solution (~ 0.5 ml) was precipitated with 50 µl of 3M sodium acetate, pH 5.2 and 1.1 ml of 100% ethanol at -80°C overnight, and the precipitated DNA was washed with 1 ml of 70% ethanol. The resulting *Sfi*I fragments were dissolved in 30 µl of 10 mM Tris-HCl, pH 8.0 and oligomerized by T4 DNA ligase (400,000 units/ml, NEB; 600 units at the beginning of the reaction and adding another 400 units after eight hours) in a 20 µl reaction with 15% polyethylene glycol (PEG) 3350 at 16°C for 16 hours. The oligomerized DNA was purified by a QIAGEN PCR Purification column to remove PEG and then resolved on a 2% low-melting agarose gel. Bar code oligomers with the size between 0.3 and 1 kb were purified from the gel as described above. The purified long bar code oligomers were ligated to *Sfi*I-digested and alkaline phosphatase (CIP)-treated pInsertion-ura4 vector [27] (purified from *dam* ^-^ and *dcm* ^-^ SCS110 cells) and transformed to DH5alpha *E. coli* (C2523H, New England Biolabs).

For bar code DNA sequencing, bacterial clones with large bar code inserts were first screened by extracting the total bacterial DNA from cells (~10^8^) with 30 µl of phenol/chloroform/isoamyl alcohol (25:24:1; vol: vol: vol) and 30 µl of 1X DNA loading dye (10 mM Tris-HCl pH 8.0, 10 mM EDTA, 50% glycerol (vol/vol), 0.01% bromphenol blue and xylene cyanole). This aqueous phase, containing the bacterial genomic DNA and bar code-containing plasmids, was analyzed by electrophoresis on a 0.7% agarose gel with an undigested vector without insert as a control. Cells bearing plasmids with large inserts compared to the empty vector control were identified, DNA was prepared and the insert size judged by restriction enzyme digestion. Plasmids with long bar code inserts were sequenced with primer TAIL-LB LOX71 (Table S6) to determine the bar code sequences. Individual bar code sequences were placed into a spreadsheet and then sorted and grouped using the data analysis functions of the software (Microsoft Excel v. 2007).

### Identification of insertion sites

Thermal Asymmetric Interlaced-PCR or TAIL-PCR [31] was used to determine the insertion sites of two of the mutants (*clg1* ^-^ and *spncrna*.*142* ^-^). TAIL-PCR uses three sequential PCR reactions where each reaction contains a mixture of degenerate primers plus a specific primer such that the first reaction includes a specific primer, the second reaction uses a second specific primer that hybridizes internal to the first primer, and the third reaction uses a specific primer internal to the second specific primer (described in detail in [27]). For this insertion library, the first PCR used the genomic DNA of the *S. pombe* insertion mutants as the template with the degenerate primers and TAIL-LB LOX71 (Table S6) as specific primer. The second PCR used 1 µl of a 2 x 10^-2^ dilution of the products from the first PCR as template with the degenerate primers and TAIL-LB2 (Table S6). The third PCR used 1 µl of a 2 x 10^-2^ dilution of the products from the second PCR as template with the degenerate primers and hsplam3 (Table S6). One or two discrete bands are produced in the second or third PCR in most cases. The products from the second or the third PCR reactions were sequenced with primer hsplam5 and hsplam7 (Table S6), respectively.

To identify the insertion site in the mutant bearing bar code 4030 (Table 1), splinkerette PCR was carried out as described [33,68]. Genomic DNA from this mutant was digested with *Spe*I and *Xba*I (which produce CTAG 5’ overhangs) and ligated to the splinkerette adaptor made by annealing oligonucleotides SPLK_A and SPLK_B_SpeI/XbaI (Table S6). This ligation product was used as the template in a first PCR reaction with primers SPLKFwd_1 and Bar code 08-4030AS (Table S6). The second PCR reaction used 1 µl of a 2 x 10^-2^ dilution of the products from the first PCR as template with primers SPLKFwd_2 and Bar code 08-4030AS, and the product was then sequenced using the SPLKFwd_2 primer (Table S6).

### Expression plasmid constructions

To test for Clg1p – Pef1p interaction by the two-hybrid assay, DNA encoding complete *clg1* ^+^ coding sequence (primers GAD_clg1_5' and Clg1_ORF_3', Table S6) or a partial *clg1* ^+^ fragment corresponding to the first 590 nucleotides of the *clg1* ^+^ ORF (primers GAD_clg1_5' and Clg1 5'+ InvU4-ASS, Table S6) was amplified by PCR and cut with *Sal*I. The resulting fragments were ligated to the vector pGAD424 (encoding the Gal4p transcription activation domain) that had been cut with *Bgl*II, blunted with T4 DNA polymerase and then digested with *Sal*I to form pGAD424-Clg1(full length) or pGAD424-Clg1(N). To generate an intron-less *pef1* ^+^ coding sequence, the second exon of *pef1* ^+^ was amplified by PCR using primers Pef1_2_exon and Pef1_ORF_3' (Table S6.) The resulting PCR product was subjected to a second PCR using primers GBD_pef1_5' (Table S6), which includes the entire first exon of *pef1* ^+^, and Pef1_ORF_3' to generate the full-length, intron-less *pef1* ^+^ coding sequence. The final PCR product was digested with *BamH* I and *Sal*I and cloned into the same sites on pGBT9 to fuse the Gal4p DNA binding domain to Pef1p.

To express N-terminally FLAG-tagged Clg1p, Cek1p or Ppk18p in fission yeast, we constructed the vector pREP41-FLAG that encodes an ATG followed by the FLAG epitope-coding sequence and a peptide linker (Gly–Gly–Ala–Ala–Ala; made by annealing oligonucleotides pREP1_ FLAG_S and pREP1_FLAG_ AS, Table S6) in the *Nde*I and *BamH* I sites on pREP41 vector [69]. The ORFs encoding Clg1p or Cek1p (primers Clg1_5’_Not I plus Clg1_3’_BamH I and Cek1_5’_Not I plus Cek1_3’_BamH I, respectively, Table S6) were generated by PCR, digested with *Not*I and *BamH* I and ligated to the same sites on pREP41-FLAG. Similarly, the ORF encoding Ppk18p was PCR amplified (primers Ppk18_5’_Not I and Ppk18_3’_Nhe I, Table S6) and digested with *Not*I and *Nhe*I, and cloned at the same sites of pREP41-FLAG. The two-hybrid and FLAG-expression constructs were verified by restriction enzyme digestion and sequencing.

### Yeast two hybrid assay

To perform the two hybrid assay to test for interaction between Clg1p and Pef1p, the pGBT9-Pef1 and pGAD424-Clg1(full length) or pGAD424-Clg1(1-590) plasmids were transformed into the budding yeast two hybrid indicator strain Y187 (*MAT*α, *ura3-52*, *his3-200*, *ade2-101*, *trp1-901*, *leu2-3*, *112*, *gal4*Δ, *met* ^*-*^, *gal80*Δ, *MEL1*, *URA3*:: *GAL1*_*UAS*_ *-GAL1*_*TATA*_ *-lacZ*; Clontech). Transformants were selected on complete medium plates without leucine and tryptophan at 30°C for 3 days. To detect the expression of the reporter gene *lacZ*, five individual colonies from each transformation were patched on plates that require both plasmids for growth and incubated at 30°C for two days. Cell patches were lifted on Whatman chromatography paper (type CHR) and submerged into liquid nitrogen for 30 seconds. The CHR paper with frozen cell patches was thawed at room temperature for three to five minutes and overlaid on another CHR paper soaked in 3 ml of X-gal-containing buffer (60 mM Na_2_HPO_4_, 40 mM NaH_2_PO_4_, 10 mM KCl, 1 mM MgSO_4_, 1 mg/ml X-gal, pH 7.0, 39 mM β-mercaptoethanol). The reaction was kept in the dark and allowed to proceed for at least one hour at room temperature.

### Protein extraction, immunoprecipitation and Western blotting

Protein expression and lysate preparation were performed as previously described [36,66,69]. Single colonies of transformed cells were inoculated into 2 ml of EMM + adenine, leucine (225 mg/l), 2% glucose, 5 µg/ml of thiamine and grown for two days at 30°C. Cells were washed with 1 ml of sterile milliQ H_2_O before being diluted into 50 ml of the same medium without thiamine at 5 x 10^5^ cells/ml and grown in an air platform shaker rotating at 220 rpm at 30°C for 24 hours to allow protein expression. Cells were then pelleted, washed with 5 ml of pre-chilled stop buffer (150 mM NaCl, 50 mM NaF, 10 mM EDTA, 1 mM sodium azide) and then with 1 ml of pre-chilled lysis buffer (25 mM MOPS pH7.2, 15 mM MgCl_2_, 15 mM EGTA, 1 mM DTT, 1% Triton-X100, 60 mM β-glycerophosphate, 15 mM p-nitrophenylphosphate, 0.1 mM sodium vanadate, 1 mM PMSF, 1X protease inhibitor cocktail (Roche)). Cells were lysed in Mini-Beadbeater-16 (Biospec) using 2-ml polypropylene screw cap tubes with 100 µl of lysis buffer and zirconia/silica beads (added to ~ 2 mm below the meniscus) with four 30-second pulses, with 1 minute on ice between each pulse. The beads were then washed with 500 µl of pre-chilled lysis buffer, centrifuged at 13,200 rpm at 4°C for 5 minutes, and the supernatant was transferred to new tubes, followed by incubation on ice for 20 minutes [36]. After a final centrifugation at 13,200 rpm at 4°C for 15 minutes, the soluble fraction was transferred to new tubes and protein concentration was determined by the Bradford method (BioRad).

For immunoprecipitation (IP), ~5 mg of protein lysate in lysis buffer was combined with 3 µg of mouse monoclonal anti-HA (F-7, Santa Cruz) or anti-FLAG (M2, Sigma) antibody and 30 µl of protein G Sepharose (GE Healthcare) in a final reaction volume of 500 µl. Sodium chloride was added to a final concentration of 150 mM in each sample. After a 4 hr incubation with rocking at 4°C, the IP samples were washed by repeated centrifugation and resuspension (3 times) in 1 ml of pre-chilled lysis buffer containing 150 mM NaCl, prior to SDS-PAGE analysis. To reduce degradation of overexpressed FLAG-Cek1p and FLAG-Ppk18p, these immunoprecipitates were denatured by incubating the washed IP samples in 1X SDS sample buffer with β-mercaptoethanol at room temperature for 20 minutes before loading the samples on the gel. Western analysis used the primary antibodies rabbit polyclonal anti-HA (Y-11, Santa Cruz) and rabbit polyclonal anti-FLAG (F7425, Sigma). Donkey anti-rabbit HRP-conjugated secondary antibody (Santa Cruz) was used in all experiments.

### DNA sequences from this work

All genomic DNA sequences discovered in this work were already present in the NCBI Genbank database. As the sequences generated in this work in confirmed existing sequences in the database, no new sequences were generated or submitted to Genbank.

## Supporting Information

Figure S1 Hypothetical survival curves of long-lived mutants with different median and maximum lifespans. (A) Long-lived mutants can be distinguished from cells with normal lifespan (the solid curve) when they are monitored in separate cultures and samples for viable cells are taken towards the end of the lifespan (e.g. the gray bar labeled “Sampling Time”). Some long-lived mutants have both longer median and maximum lifespans (the dashed curve), while others only have extended median lifespans (the dotted curve). (B) In a CLS assay of pooled random mutants, the initial proportion of the desired long-lived mutants can be very small (e.g. 1/10^6^ of total population in this figure). Viable cells in samples taken from the culture near the end of the lifespan (the gray bar) have a larger proportion of long-lived mutants (e.g. ~ 1/10^2^ in this example), and these long-lived mutants can be distinguished from the cells with normal lifespans if each mutant bears a unique bar code, as described in the main text.
(TIF)

Figure S2 The CLS of the pool of bar-coded *S. pombe* insertion mutants. A pool of 3600 mutants were aged in a single flask containing 240 ml of SD + 3% glucose liquid medium. The CFU/ml was monitored for 15 days. On day 14, colonies from 600 surviving cells were collected for bar code sequencing and subsequent analysis.
(TIF)

Figure S3 Survival curves of the most frequently isolated surviving mutants. The original isolates of the three most frequently isolated mutants (shown in Table 1) were assayed for lifespan where each strain was analyzed in individual cultures. All assays were performed in duplicate at the same time in parallel with the wild type controls. For clarity, survival curves are shown with one mutant and the wild type strain. (A) The original *clg1::bar code-ura4* mutant had a longer lifespan than wild type cells. (B) The 28S rRNA gene insertion mutant had a longer lifespan than wild type cells. (C) The original *spncrna.142* mutant had a survival curve that overlaps that of the wild type strain. When assayed individually in culture, this insertion mutant did not show the extended lifespan suggested by its increased bar code frequency in the culture of 3600 mutants. The reasons that the high frequency of the bar code from this mutant was present in the final pool of surviving cells are unknown and may reflect a difference between the environments of the individual culture and a culture of mixed mutants. Identification of mutants with increased longevity in cultures of mixed mutants but not in individual cultures has also been observed in *S. cerevisiae* [1].
(TIF)

Figure S4 The schematic representation of the three *S. pombe* Pef1p-associated cyclins. (A) The cyclin Clg1p was identified in our screen for long-lived mutants and shown to interact with the Cdk Pef1p. The numbers above the white and black boxes are the number of amino acids. The cyclin domain in each protein is shown as the black box. The black arrowhead represents the location of the insertion mutation. (B) The previously identified Pas1p cyclin that associates with Pef1p [2]. (C) The reading frame Spbc20f10.10p was identified as having a cyclin domain and is shown to associate with Pef1p in the main text, and is given the new common name Psl1p.
(TIF)

Figure S5 The individual survival curves of *pef1∆* and *pef1∆ clg1∆* compared to wild type cells. These graphs replot the data from Figure 3 with error bars for a direct comparison of each mutant with wild type cells. The same wild type survival curve is used in panels A and B. (A) The *pef1∆* mutant had an extended lifespan compared to wild type cells. (B) The *clg1∆ pef1∆* double mutant had a longer CLS than wild type cells. (C) The *pef1∆, clg1∆* and *clg1∆ pef1∆* mutants had very similar lifespans, consistent with Pef1p and Clg1p acting in the same pathway. Statistical comparisons between the different curves are presented in Table S5.
(TIF)

Figure S6 The individual survival curves of *pas1∆* and *psl1∆* compared to wild type cells. These graphs replot the data from Figure 4B with error bars for a direct comparison of each mutant with wild type cells. The same wild type survival curve is used in panels A and B. (A) The *psl1∆* mutant had a shorter lifespan compared to wild type cells. (B) The *pas1∆* mutant had the same lifespan as wild type cells. Statistical comparisons between the different curves are presented in Table S5.
(TIF)

Figure S7 The individual survival curves of *clg1∆, cek1∆* and *clg1∆ cek1∆* compared to wild type cells. These graphs replot the data from Figure 5 with error bars for a direct comparison of each mutant strain with the wild type one. The same wild type survival curve is used in panels A, B and C. The *clg1∆* mutant had a longer lifespan compared to wild type cells (A), while the *cek1∆* mutant had a lifespan very similar to the wild type strain (B). The *clg1∆ cek1∆* double mutant had a lifespan similar to the wild type strain (C) and the *cek1∆* single mutant (D). These data suggest that Clg1p and Cek1p act in the same genetic pathway to control lifespan. Statistical comparisons between the different curves are presented in Table S5.
(TIF)

Figure S8 The individual survival curves of *clg1∆, ppk18∆* and *clg1∆ ppk18∆* compared to wild type cells. These graphs replot the data from Figure 6 with error bars for a direct comparison of each mutant with wild type cells. The same wild type survival curve is used in panels A, B and C. The *clg1∆* mutant had a longer lifespan compared to wild type cells (A), while the *ppk18∆* mutant had a lifespan much shorter than the wild type strain (B). The *clg1∆ ppk18∆* double mutant had a lifespan shorter than the wild type strain (C) which was intermediate compared to the *clg1∆* and *ppk18∆* single mutants (D). These data suggest that Clg1p and Ppk18p act in different genetic pathways to control lifespan. Statistical comparisons between the different curves are presented in Table S5.
(TIF)

Figure S9 The survival curves of *psl1∆* double mutants compared to wild type cells. (A) The lifespans of *psl1∆ pef1∆, psl1∆ clg1∆* and *psl1∆ cek1∆* double mutant cells compared to wild type cells. Loss of Pef1p from *psl1∆* cells extends lifespan similar to that of the *pef1∆* single mutant (Figure 3), loss of Clg1p from *psl1∆* cells results in a wild type lifespan and loss of Cek1p from *psl1∆* cells has a small effect on lifespan. (B) An enlarged graph showing the smaller differences between the *psl1∆, cek1∆, psl1∆ cek1∆* cells and wild cells. While the lifespan of *psl1∆* cells is shorter than the *cek1∆* and *psl1∆ cek1∆* cells, the lifespans of the *cek1∆* and *psl1∆ cek1∆* cells is not significantly different (Table S5).
(TIF)

Figure S10 Oxidative and heat shock stress sensitivity of *clg1*Δ, *pef1*Δ, *clg1*Δ *pef1*Δ and *cek1*Δ mutant cells. Cells from day 1 cultures of a CLS assay were collected and washed with sterile milliQ H_2_O followed by exposure to different concentrations of H_2_O_2_ at 30°C for 1.5 hours at the density of 10^7^ cells/ml. Treated cells were washed with sterile milliQ H_2_O and 10-fold serially diluted in sterile H_2_O. For heat shock stress, cells were similarly washed and resuspended in pre-heated sterile milliQ water and incubated in a 55°C water bath for 3 or 10 minutes and then put on ice for 2 minutes. The 0 min heat shock control cells were resuspended in 55°C pre-warmed sterile milliQ water and immediately chilled on ice for two minutes and 10-fold serially diluted in sterile H_2_O. Five µl of each dilution was spotted on YES plates and grown at 30°C for 5 days. The assays were done twice in duplicate and representative results are shown.
(TIF)

Table S1 Bar code sequencing of the initial mutant pool. (DOC)

Table S2 *S. pombe* proteins with cyclin_N domain PF00134. (DOC)

Table S3 Potential *S. pombe* homologs of *S. cerevisiae* Rim15p by sequence homology and conserved protein domains. (DOC)

Table S4 Potential homologs of *S. pombe* Clg1p, Pef1p and Cek1p in budding yeast, humans, flies and worms. (DOC)

Table S5 Wilcoxon matched-pairs signed rank test of CLS experiments. (DOC)

Table S6 Oligonucleotides used in this study. (DOC)

References S1 (DOCX)
