# Supplementary figures and images for: Identification of a Lifespan Extending Mutation in the Schizosaccharomyces pombe Cyclin Gene clg1 + by Direct Selection of Long-Lived Mutants
Source: PLoS One. 2013 Jul 9;8(7):e69084. doi: 10.1371/journal.pone.0069084 (PMC3711543; doi:10.1371/journal.pone.0069084)

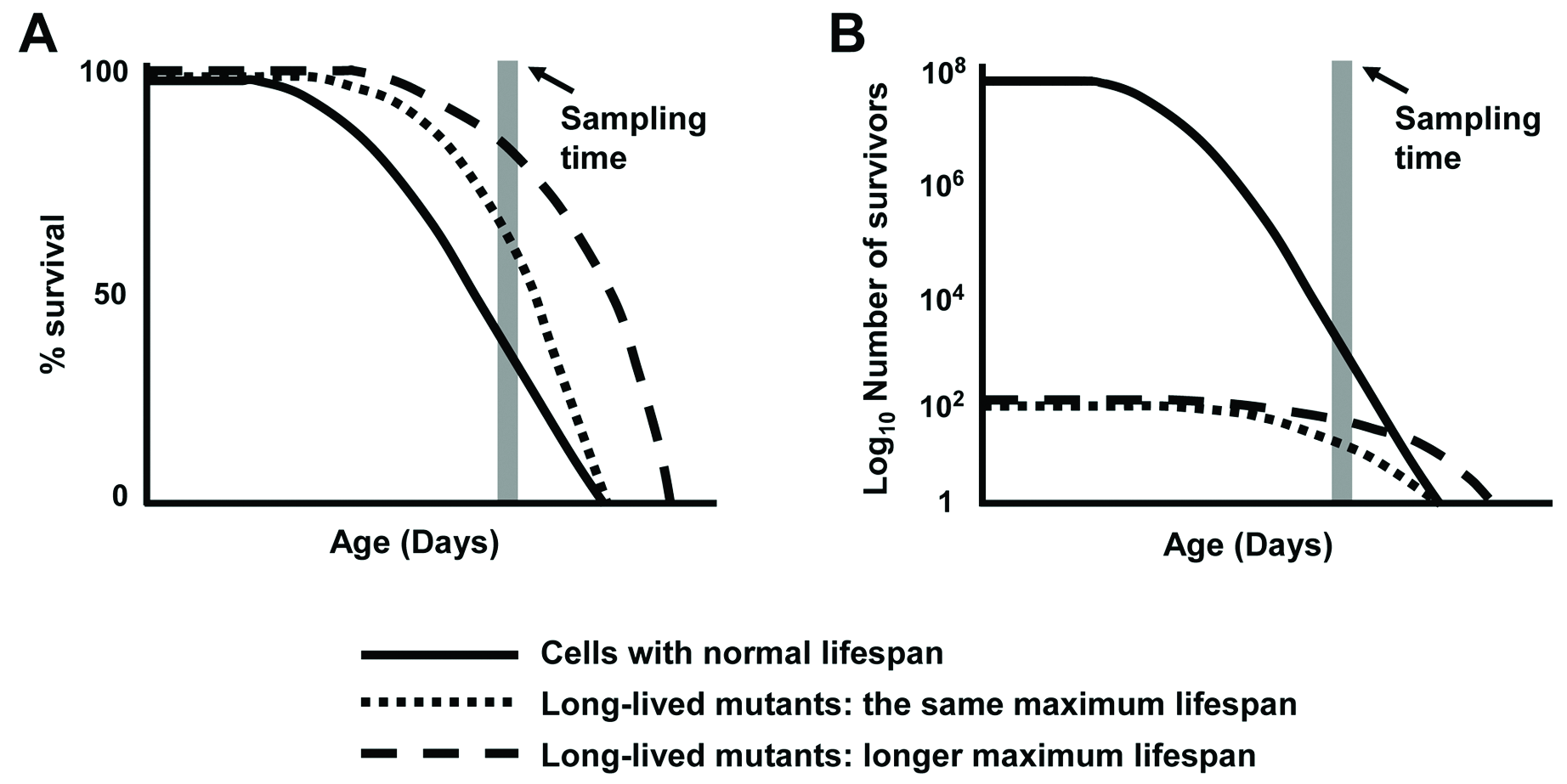

Supplement: Figure S1 — (A) Long-lived mutants can be distinguished from cells with normal lifespan (the solid curve) when they are monitored in separate cultures and samples for viable cells are taken towards the end of the lifespan (e.g. the gray bar labeled “Sampling Time”). Some long-lived mutants have both longer median and maximum lifespans (the dashed curve), while others only have extended median lifespans (the dotted curve). (B) In a CLS assay of pooled random mutants, the initial proportion of the desired long-lived mutants can be very small (e.g. 1/106 of total population in this figure). Viable cells in samples taken from the culture near the end of the lifespan (the gray bar) have a larger proportion of long-lived mutants (e.g. ~ 1/102 in this example), and these long-lived mutants can be distinguished from the cells with normal lifespans if each mutant bears a unique bar code, as described in the main text. (TIF) [file pone.0069084.s001.tif]

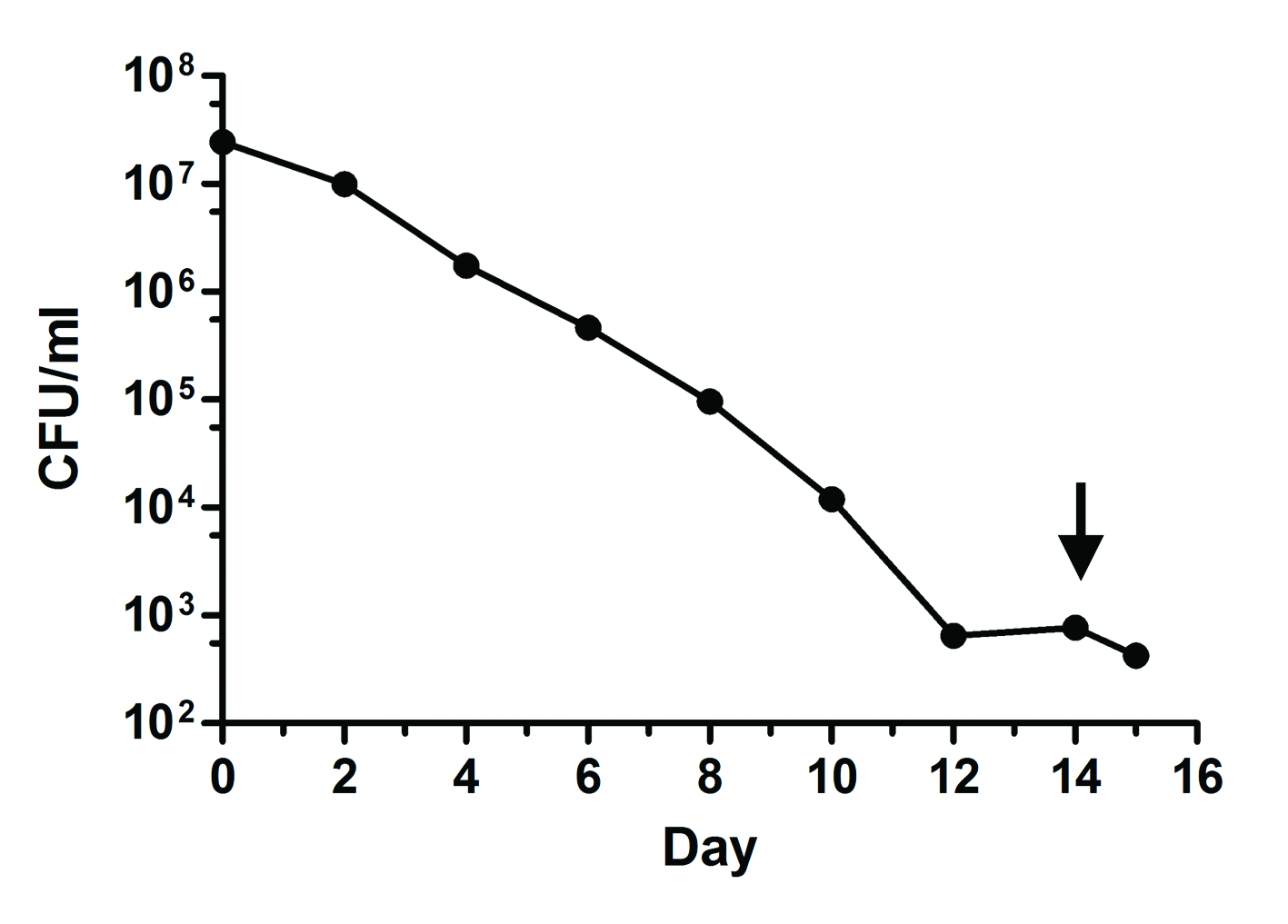

Supplement: Figure S2 — A pool of 3600 mutants were aged in a single flask containing 240 ml of SD + 3% glucose liquid medium. The CFU/ml was monitored for 15 days. On day 14, colonies from 600 surviving cells were collected for bar code sequencing and subsequent analysis. (TIF) [file pone.0069084.s002.tif]

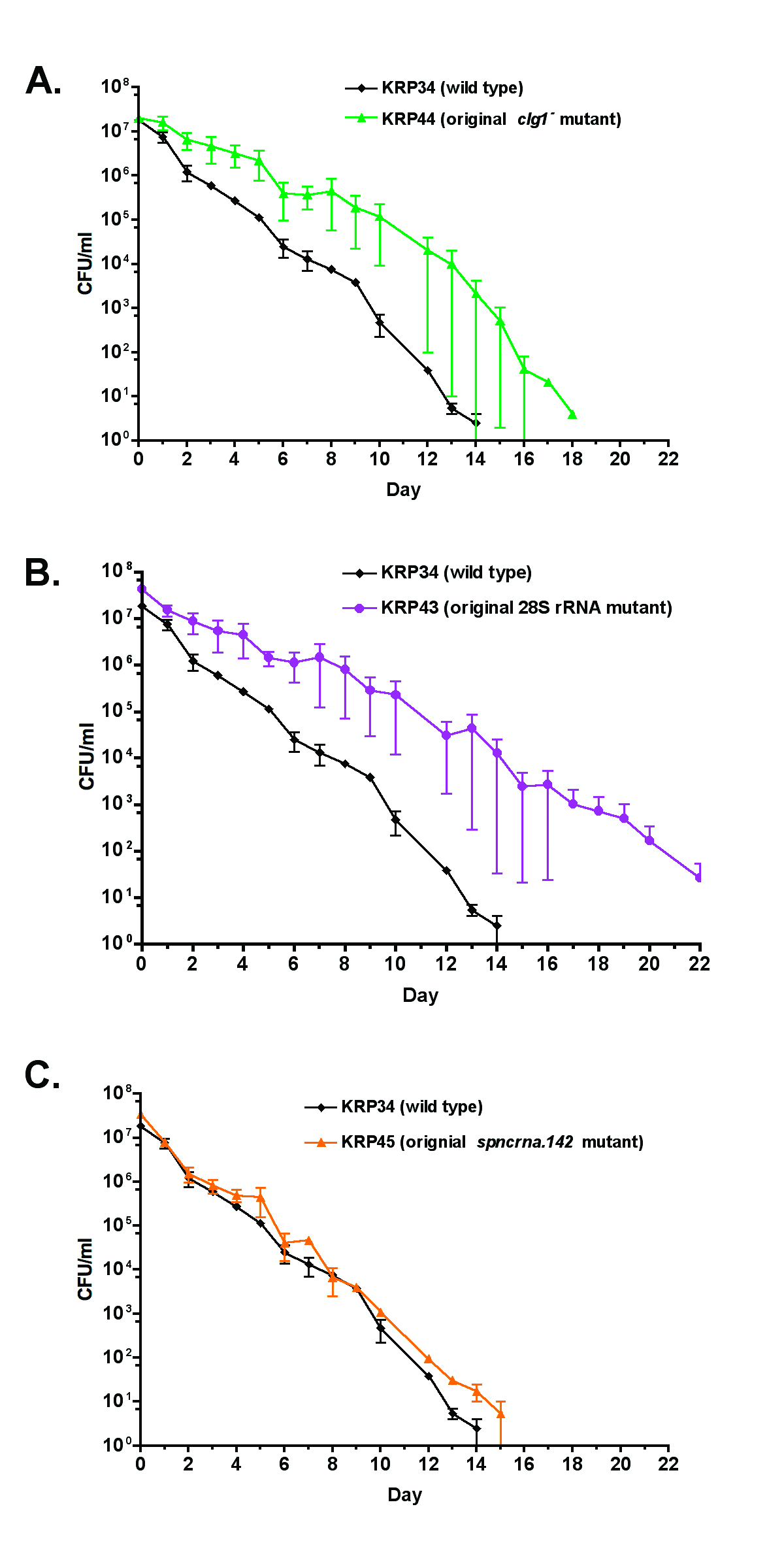

Supplement: Figure S3 — The original isolates of the three most frequently isolated mutants (shown in Table 1) were assayed for lifespan where each strain was analyzed in individual cultures. All assays were performed in duplicate at the same time in parallel with the wild type controls. For clarity, survival curves are shown with one mutant and the wild type strain. (A) The original clg1::bar code-ura4 mutant had a longer lifespan than wild type cells. (B) The 28S rRNA gene insertion mutant had a longer lifespan than wild type cells. (C) The original spncrna.142 mutant had a survival curve that overlaps that of the wild type strain. When assayed individually in culture, this insertion mutant did not show the extended lifespan suggested by its increased bar code frequency in the culture of 3600 mutants. The reasons that the high frequency of the bar code from this mutant was present in the final pool of surviving cells are unknown and may reflect a difference between the environments of the individual culture and a culture of mixed mutants. Identification of mutants with increased longevity in cultures of mixed mutants but not in individual cultures has also been observed in S. cerevisiae [1]. (TIF) [file pone.0069084.s003.tif]

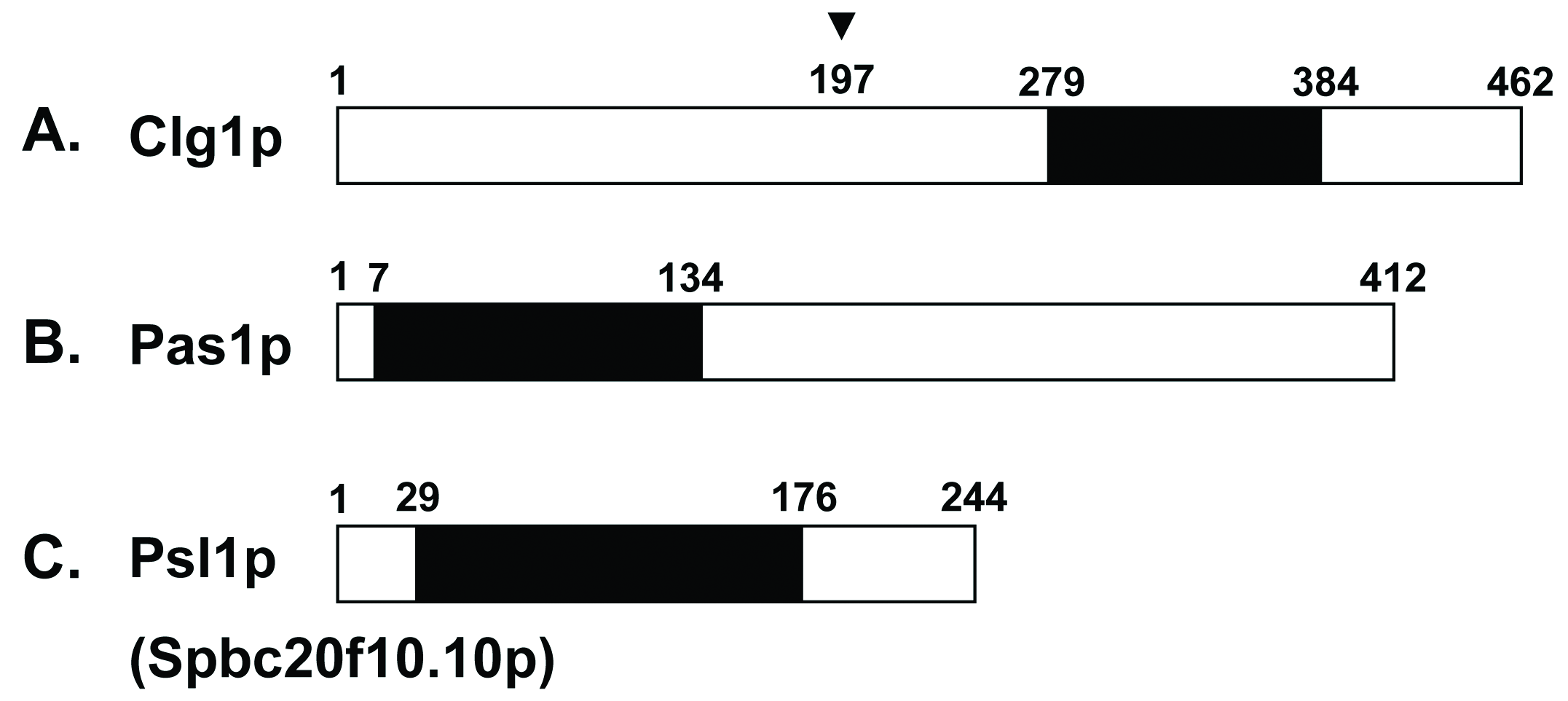

Supplement: Figure S4 — (A) The cyclin Clg1p was identified in our screen for long-lived mutants and shown to interact with the Cdk Pef1p. The numbers above the white and black boxes are the number of amino acids. The cyclin domain in each protein is shown as the black box. The black arrowhead represents the location of the insertion mutation. (B) The previously identified Pas1p cyclin that associates with Pef1p [2]. (C) The reading frame Spbc20f10.10p was identified as having a cyclin domain and is shown to associate with Pef1p in the main text, and is given the new common name Psl1p. (TIF) [file pone.0069084.s004.tif]

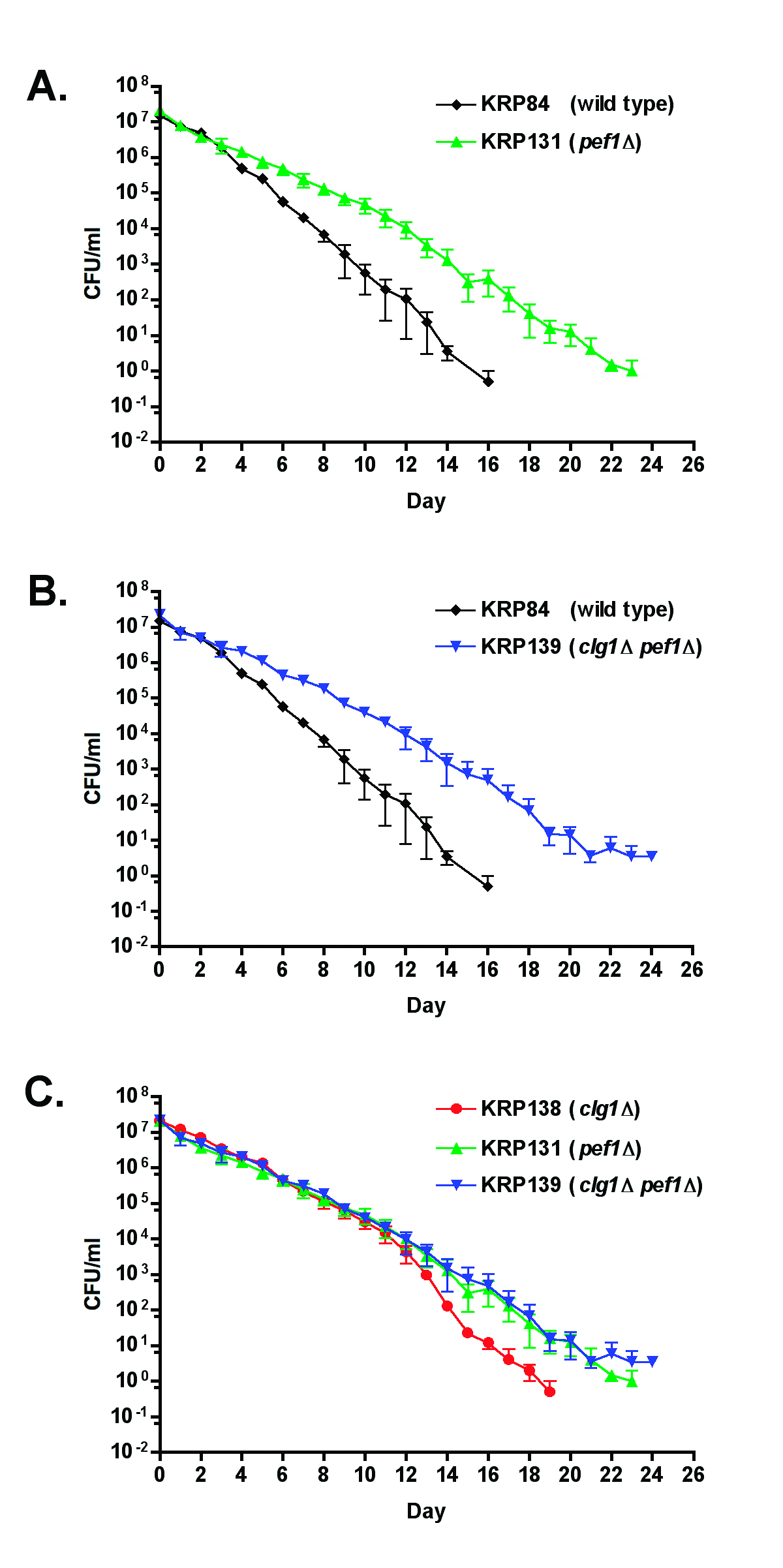

Supplement: Figure S5 — These graphs replot the data from Figure 3 with error bars for a direct comparison of each mutant with wild type cells. The same wild type survival curve is used in panels A and B. (A) The pef1∆ mutant had an extended lifespan compared to wild type cells. (B) The clg1∆ pef1∆ double mutant had a longer CLS than wild type cells. (C) The pef1∆, clg1∆ and clg1∆ pef1∆ mutants had very similar lifespans, consistent with Pef1p and Clg1p acting in the same pathway. Statistical comparisons between the different curves are presented in Table S5. (TIF) [file pone.0069084.s005.tif]

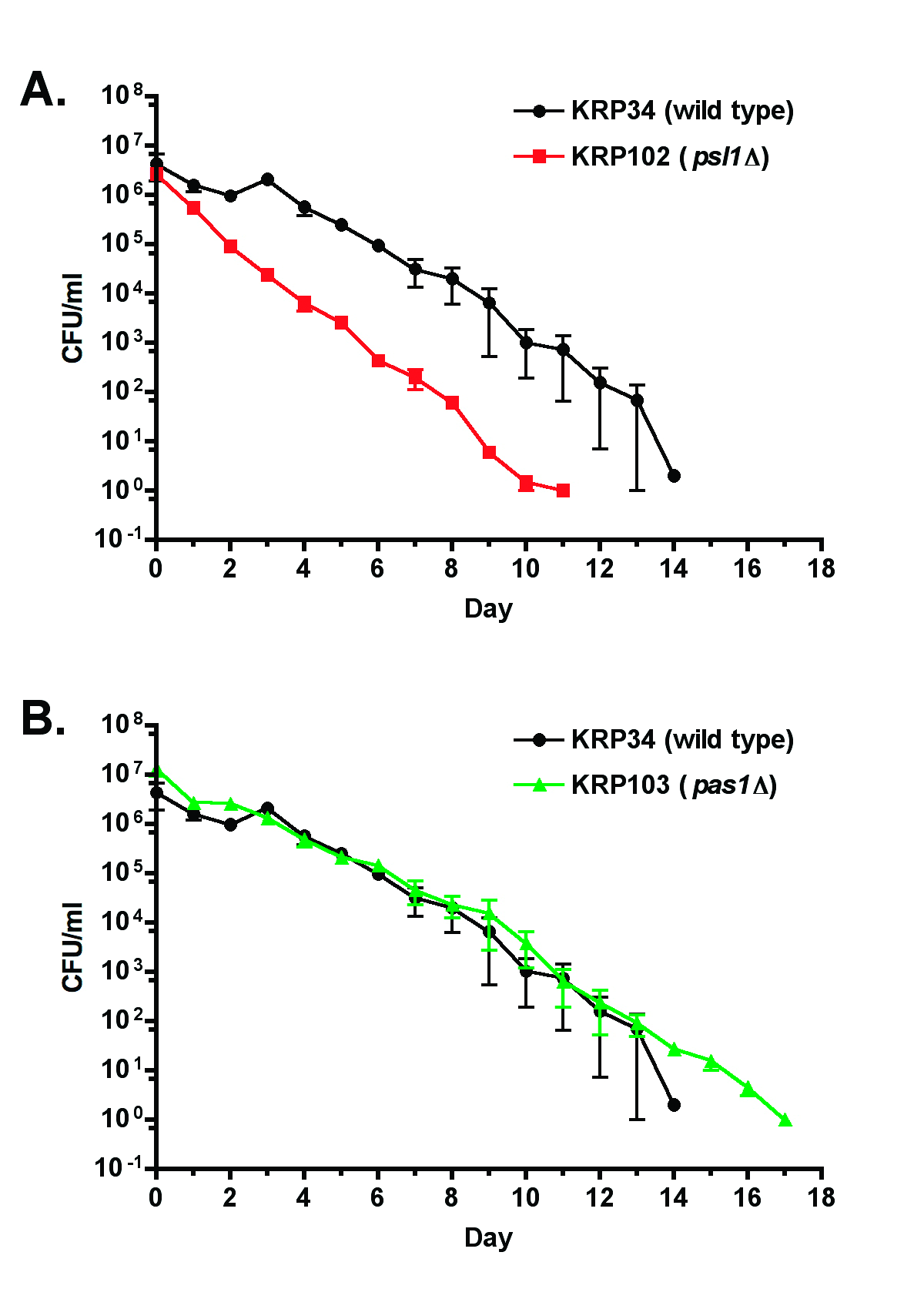

Supplement: Figure S6 — These graphs replot the data from Figure 4B with error bars for a direct comparison of each mutant with wild type cells. The same wild type survival curve is used in panels A and B. (A) The psl1∆ mutant had a shorter lifespan compared to wild type cells. (B) The pas1∆ mutant had the same lifespan as wild type cells. Statistical comparisons between the different curves are presented in Table S5. (TIF) [file pone.0069084.s006.tif]

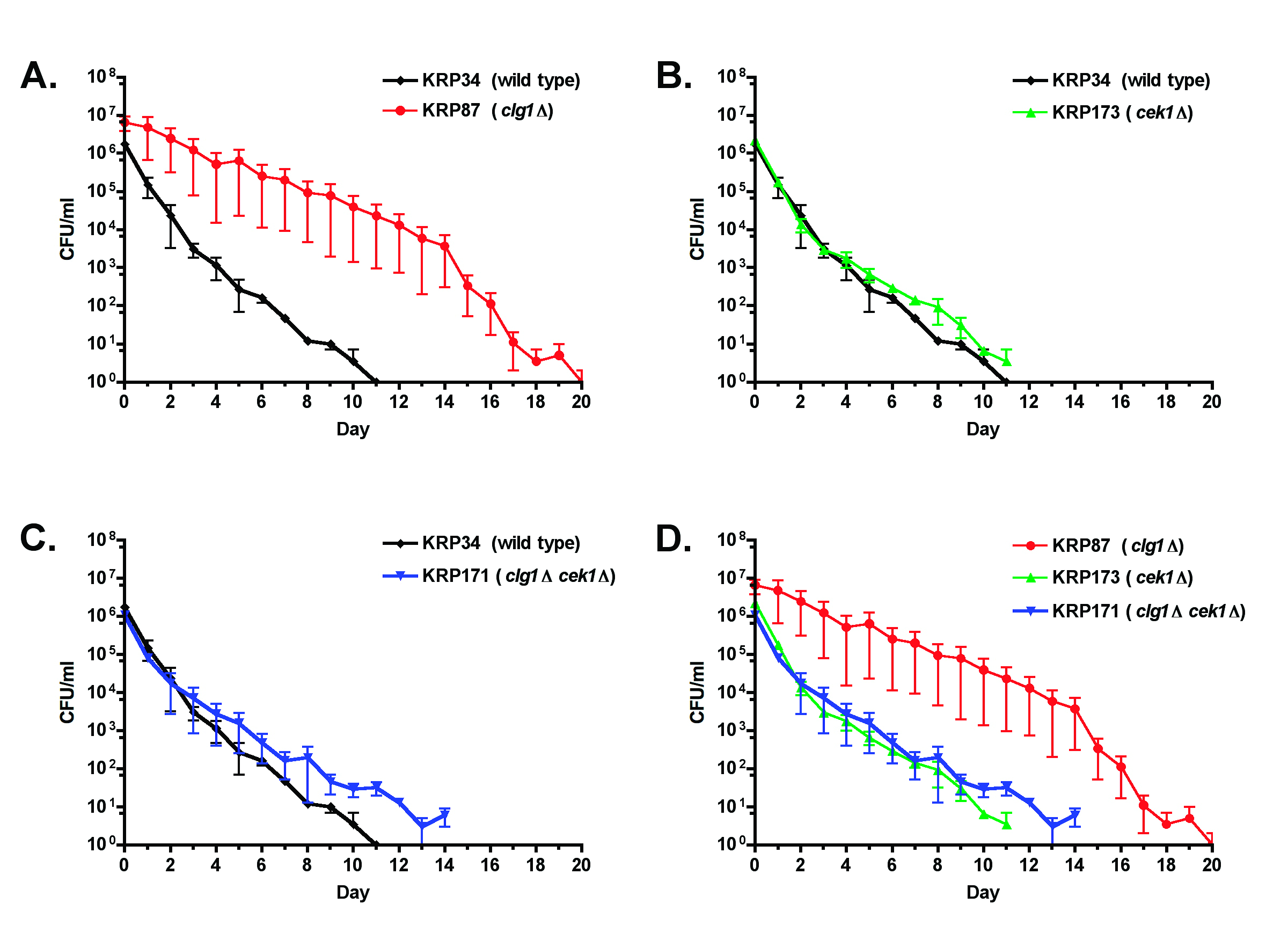

Supplement: Figure S7 — These graphs replot the data from Figure 5 with error bars for a direct comparison of each mutant strain with the wild type one. The same wild type survival curve is used in panels A, B and C. The clg1∆ mutant had a longer lifespan compared to wild type cells (A), while the cek1∆ mutant had a lifespan very similar to the wild type strain (B). The clg1∆ cek1∆ double mutant had a lifespan similar to the wild type strain (C) and the cek1∆ single mutant (D). These data suggest that Clg1p and Cek1p act in the same genetic pathway to control lifespan. Statistical comparisons between the different curves are presented in Table S5. (TIF) [file pone.0069084.s007.tif]

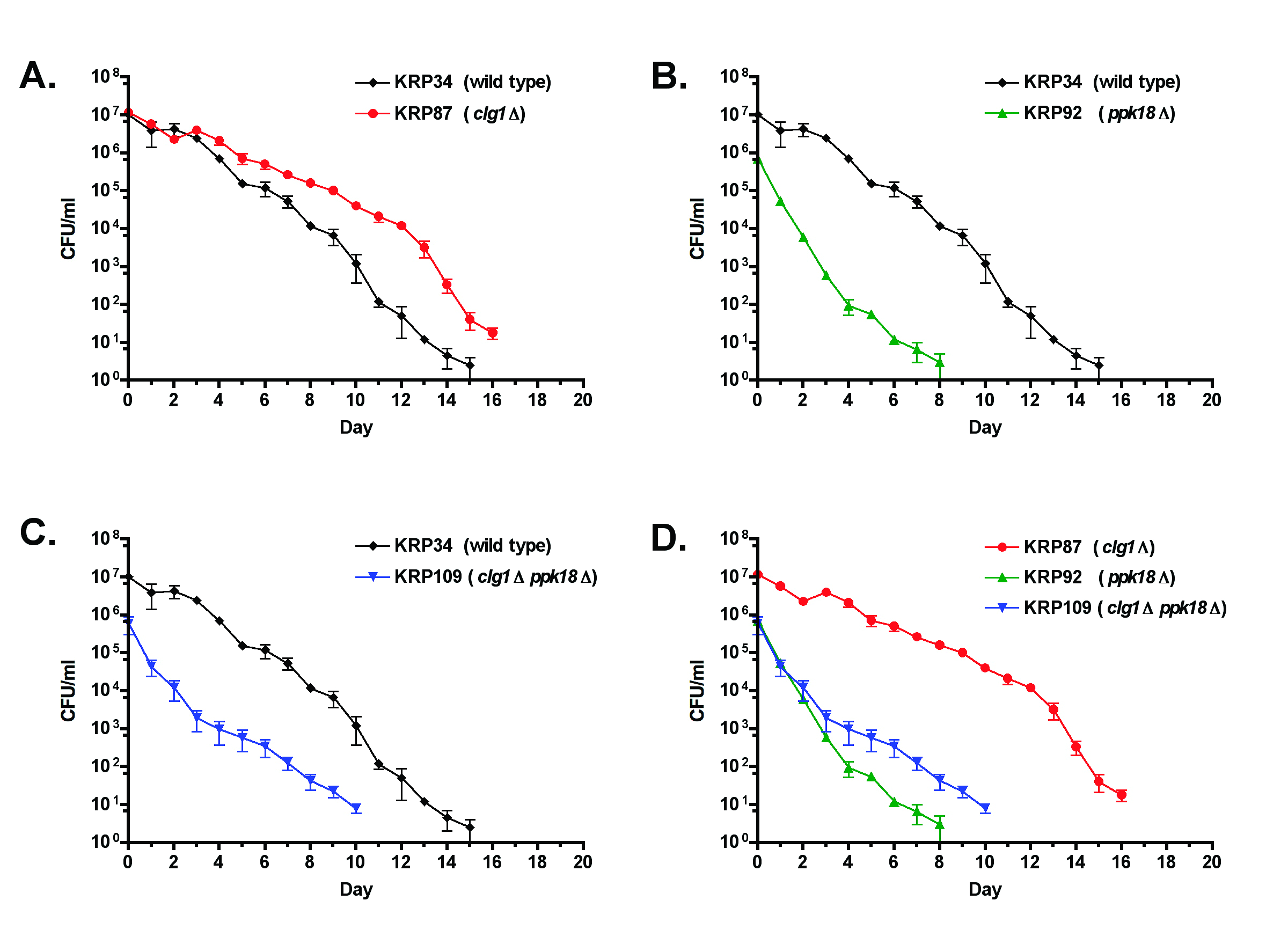

Supplement: Figure S8 — These graphs replot the data from Figure 6 with error bars for a direct comparison of each mutant with wild type cells. The same wild type survival curve is used in panels A, B and C. The clg1∆ mutant had a longer lifespan compared to wild type cells (A), while the ppk18∆ mutant had a lifespan much shorter than the wild type strain (B). The clg1∆ ppk18∆ double mutant had a lifespan shorter than the wild type strain (C) which was intermediate compared to the clg1∆ and ppk18∆ single mutants (D). These data suggest that Clg1p and Ppk18p act in different genetic pathways to control lifespan. Statistical comparisons between the different curves are presented in Table S5. (TIF) [file pone.0069084.s008.tif]

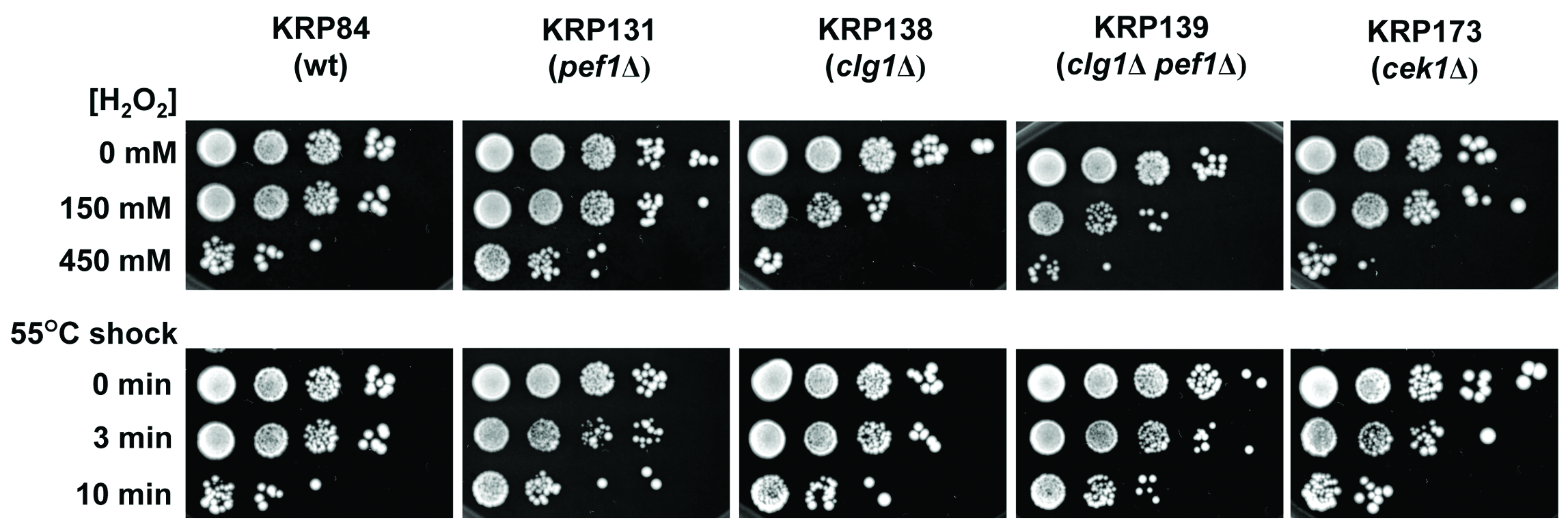

Supplement: Figure S10 — Cells from day 1 cultures of a CLS assay were collected and washed with sterile milliQ H2O followed by exposure to different concentrations of H2O2 at 30°C for 1.5 hours at the density of 107 cells/ml. Treated cells were washed with sterile milliQ H2O and 10-fold serially diluted in sterile H2O. For heat shock stress, cells were similarly washed and resuspended in pre-heated sterile milliQ water and incubated in a 55°C water bath for 3 or 10 minutes and then put on ice for 2 minutes. The 0 min heat shock control cells were resuspended in 55°C pre-warmed sterile milliQ water and immediately chilled on ice for two minutes and 10-fold serially diluted in sterile H2O. Five µl of each dilution was spotted on YES plates and grown at 30°C for 5 days. The assays were done twice in duplicate and representative results are shown. (TIF) [file pone.0069084.s010.tif]
