# Supplementary material for: Identification of a Lifespan Extending Mutation in the Schizosaccharomyces pombe Cyclin Gene clg1 + by Direct Selection of Long-Lived Mutants
Source: PLoS One. 2013 Jul 9;8(7):e69084. doi: 10.1371/journal.pone.0069084 (PMC3711543; doi:10.1371/journal.pone.0069084)
Supplement: Table S1 — (DOC) [file pone.0069084.s011.doc]

| **Table S1.** Barcode sequencing of the initial mutant pool. | | |
| --- | --- | --- |
| **Frequencies of barcode** | **Types of barcode^a^** | **% of all mutants examined** |
| 1 | 117 | 0.69 |
| 2 | 15 | 0.18 |
| 3 | 6 | 0.11 |
| 4 | 1 | 0.02 |

**^a^** The total number of sequenced barcodes is 169.
