# Supplementary material for: Identification of a Lifespan Extending Mutation in the Schizosaccharomyces pombe Cyclin Gene clg1 + by Direct Selection of Long-Lived Mutants
Source: PLoS One. 2013 Jul 9;8(7):e69084. doi: 10.1371/journal.pone.0069084 (PMC3711543; doi:10.1371/journal.pone.0069084)
Supplement: Table S2 — (DOC) [file pone.0069084.s012.doc]

| **Table S2.** *S. pombe* proteins with cyclin_N domain PF00134**^a^** | | |
| --- | --- | --- |
| **Protein** | **Description/function** | **Reference** |
| Crs1p | Up-regulated in meiosis; required for normal meiotic nuclear division | [3,4] |
| Lsc1p | Phosphorylates RNA polymerase II C-terminal domain; regulate cytokinesis | [5,6] |
| Mcs2p | Suppresses mitotic defects of the *cdc2-3w weel-50* double mutant | [7,8] |
| Puc1p | Regulates exit from mitosis | [9,10] |
| Srb11p | Negatively regulates RNA polymerase II-dependent transcription | [11] |
| Spac1296.05c | Inferred from homology to cyclins | [12] |
| Cig1p**^b^** | B-type cyclin; regulates G_1_/S transition of mitotic cell cycle | [13,14] |
| Cig2p**^b^** | B-type cyclin; regulates G_1_/S transition of mitotic cell cycle | [15,16] |
| Cdc13p**^b^** | B-type cyclin; regulates G_2_/M transition of mitotic cell cycle; required for maintenance of quiescence; | [17,18,19] |
| Pch1p**^b^** | Involved in mRNA capping | [20,21] |
| Rem1p**^b^** | Required for proper progression through meiosis | [22] |

**^a^** As of August, 2011

**^b^** Also contain a cyclin_C domain (PF02984)
