# Supplementary material for: Identification of a Lifespan Extending Mutation in the Schizosaccharomyces pombe Cyclin Gene clg1 + by Direct Selection of Long-Lived Mutants
Source: PLoS One. 2013 Jul 9;8(7):e69084. doi: 10.1371/journal.pone.0069084 (PMC3711543; doi:10.1371/journal.pone.0069084)
Supplement: Table S3 — (DOC) [file pone.0069084.s013.doc]

| **Table S3.** Potential *S. pombe* homologs of *S. cerevisiae* Rim15p by sequence homology and conserved protein domains. | | | | | | |
| --- | --- | --- | --- | --- | --- | --- |
| **Protein** | **PAS domain^a^** | **Kinase insert^b^** | **Max score^c^** | **Total score^c^** | **Query coverage^c^** | **E-value^c^** |
| *S. cerevisiae* Rim15p | Yes | Yes | - | - | - | - |
| *S. pombe* Cek1p | Yes | Yes | 231 | 547 | 50% | 7e-63 |
| *S. pombe* Ppk18p | Yes | Yes | 212 | 551 | 59% | 1e-56 |

**^a^** Reference [23]

**^b^** Reference [24,25]; the kinase inserts between subdomain VII and VIII were also identified in a domain search on Pfam 25.0 database. The insert is 190 aa in Rim15p and 120 aa in Cek1p and Ppk18p.

**^c^** Protein blast search in NCBI using the dataset “non-redundant protein sequences” including organism “*Schizosaccharomyces pombe* 972h^-^ (taxid: 284812)” sequences only (as of November, 2011).
