# Supplementary material for: Identification of a Lifespan Extending Mutation in the Schizosaccharomyces pombe Cyclin Gene clg1 + by Direct Selection of Long-Lived Mutants
Source: PLoS One. 2013 Jul 9;8(7):e69084. doi: 10.1371/journal.pone.0069084 (PMC3711543; doi:10.1371/journal.pone.0069084)
Supplement: Table S4 — (DOC) [file pone.0069084.s014.doc]

| **Table S4.** Potential homologs of *S. pombe* Clg1p, Pef1p and Cek1p in budding yeast, humans, flies and worms. | | | |
| --- | --- | --- | --- |
| **Organism** | **Cyclin** | **Cdk** | **Downstream kinase** |
| *S. pombe* | Clg1p | Pef1p | Cek1p |
| *S. cerevisiae* | Clg1p^a^, Pho80p^b^ | Pho85p^a^ | Rim15p^a^ |
| *H. sapiens* | CNPPD1^a^ | Cdk5^c^ | LATS^a^, MAST^a^ |
| *D. melanogaster* | CG40191^d^ | Cdk5^c^, Cdc2^a^ | LATS^a^ |
| *C. elegans* | F09G2.2^d^ | Cdk5^c^, Cdk1^a^ | WarTS^a^, kin-4^a^ |

**^a^** Based on sequence homology to *S. pombe* proteins.

**^b^** Based on the hypothesized functional similarity.

**^c^** Based on functional complementation of rodent Cdk5 in *S. cerevisiae* *pho85*Δ mutants.

**^d^** Based on sequence homology to human CNPPD1.
