# Supplementary material for: Identification of a Lifespan Extending Mutation in the Schizosaccharomyces pombe Cyclin Gene clg1 + by Direct Selection of Long-Lived Mutants
Source: PLoS One. 2013 Jul 9;8(7):e69084. doi: 10.1371/journal.pone.0069084 (PMC3711543; doi:10.1371/journal.pone.0069084)
Supplement: Table S5 — (DOC) [file pone.0069084.s015.doc]

| **Table S5.** Wilcoxon matched-pairs signed rank test of CLS experiments | | | | |
| --- | --- | --- | --- | --- |
| **Figure** | **Experiment** | **P value** | **Sum of signed ranks** | **Effectiveness of pairing^a^** |
| 2C | wt vs *clg1*::barcode-*ura4*^+^ | 0.0001 | -105.0 | 0.9956 |
|  |  |  |  |  |
| 2D | wt vs *clg1*Δ | 0.0003 | -153.0 | 0.9975 |
|  |  |  |  |  |
| 3C | wt vs *clg1*Δ | 0.0003 | -153.0 | 0.9975 |
|  | wt vs *pef1*Δ | 0.0045 | -121.0 | 1.0000 |
|  | wt vs *clg1*Δ *pef1*Δ | 0.0029 | -127.0 | 0.9975 |
|  | *clg1*Δ vs *clg1*Δ *pef1*Δ | 0.4441 | -42.00 | 1.0000 |
|  | *pef1*Δ vs *clg1*Δ *pef1*Δ | 0.1128 | -112.0 | 0.9983 |
|  |  |  |  |  |
| 4B | wt vs *pas1*Δ | 0.1876 | -48.00 | 0.9893 |
|  | wt vs *psl1*Δ | 0.0005 | 78.00 | 0.9790 |
|  |  |  |  |  |
| 5 | wt vs *clg1*Δ | 0.0005 | -78.00 | 0.9930 |
|  | wt vs *cek1*Δ | 0.0640 | -48.00 | 1.0000 |
|  | wt vs *clg1*Δ *cek1*Δ | 0.6772 | -12.00 | 0.9860 |
|  | *clg1*Δ vs *cek1*Δ | 0.0002 | 91.00 | 0.9945 |
|  | *clg1*Δ vs *clg1*Δ *cek1*Δ | 0.0001 | 120.0 | 0.9857 |
|  | *cek1*Δ vs *clg1*Δ *cek1*Δ | 0.1677 | -41.00 | 0.9890 |
|  |  |  |  |  |
| 6 | wt vs *clg1*Δ | 0.0077 | -104.0 | 0.9912 |
|  | wt vs *ppk18*Δ | 0.0020 | 55.00 | 0.9879 |
|  | wt vs *clg1*Δ *ppk18*Δ | 0.0010 | 66.00 | 0.9909 |
|  | *clg1*Δ vs *clg1*Δ *ppk18*Δ | 0.0010 | 66.00 | 0.9909 |
|  | *ppk18*Δ vs *clg1*Δ *ppk18*Δ | 0.0078 | -36.0 | 1.0000 |
|  |  |  |  |  |
| S8 | wt vs *psl1*Δ *pef1*Δ | <0.0001 | -286 | 0.9524 |
|  | wt vs *psl1*Δ *clg1*Δ | 0.4212 | 30.00 | 0.9379 |
|  | wt vs *psl1*Δ *cek1*Δ | 0.0001 | 105 | 0.9456 |
|  | *psl1*Δ vs *psl1*Δ *cek1*Δ | 0.0024 | -72.00 | 0.8275 |
|  | *cek1* vs *psl1*Δ *cek1*Δ | 0.0906 | -55.00 | 0.8919 |
|  | wt vs *cek1*Δ | 0.0002 | 102 | 0.9346 |
|  | wt vs *psl1*Δ | 0.0002 | 102 | 0.8222 |
|  | *psl1*Δ vs *cek1*Δ | 0.0803 | -51.00 | 0.7722 |

**^a^** Spearman correlation coefficient (rs) for effectiveness of pairing.  Pairing was effective in all comparisons (P value for all pairings are smaller than 0.0001).
