# Supplementary material for: Identification of a Lifespan Extending Mutation in the Schizosaccharomyces pombe Cyclin Gene clg1 + by Direct Selection of Long-Lived Mutants
Source: PLoS One. 2013 Jul 9;8(7):e69084. doi: 10.1371/journal.pone.0069084 (PMC3711543; doi:10.1371/journal.pone.0069084)
Supplement: Table S6 — (DOC) [file pone.0069084.s016.doc]

| **Table S6.** Oligonucleotides used in this study. | |
| --- | --- |
| **Name** | **Sequence** |
| hsplam3 | AAGGGAAAAGTCGACTCTCCGTGACGACTTATAAAAGCCCAGGGGCAAG |
| hsplam5 | GGATAACGGCTAACGGTGTACGTCAGCCCGGAAAAGTGCATATCCAG |
| hsplam6 | GTACCTAATATTTTCACGATGTTCTGCTGGATATGCACTTTTCCGGGCTG |
| hsplam7 | CATCGTGAAAATATTAGGTACTGTAAAAGCGGTGCCAGTCGGCATAC |
| TAIL-LB2 | CTCCATTAAGTAACAAATTCCTATTTAGAGAAAGAATGCTGAGTA |
| TAIL-LB LOX71 | AGCCAGTGGATAACTTCGTATAATGTATGCTATACGAACGGTA |
| TAIL AD1**^a^** | NGTCGASWGANAWGAA |
| TAIL AD2**^a^** | TGWGNAGSANCASAGA |
| TAIL AD3**^a^** | AGWGNAGWANCAWAGG |
| TAIL AD4**^a^** | STTGNTASTNCTNTGC |
| TAIL AD5**^a^** | NTCGASTWTSGWGTT |
| TAIL AD6**^a^** | WGTGNAGWANCANAGA |
| BarcodePCR(888r) | CACGACATGTGCAGAGATGCCGACGAAGCA |
| SPLK A | GAAGAGTAACCGTTGCTAGGAGAGACCGTGGCTGAATGAGACTGGTGT CGACACTAGTGG |
| SPLK B Spe I Xba I | CTAGCCACTAGTGTCGACACCAGTCTCTAATTTTTTTTTTCAAAAAAA |
| SPLKFwd 1 | GAAGAGTAACCGTTGCTAGGAGAGACC |
| SPLKFwd 2 | GTGGCTGAATGAGACTGGTGTCGAC |
| Barcode 08-4030AS | AATGATAGCAATAGACAAATATAAAGCAACAAGAACAATGA |
| GAD_clg1_5' | GGCGTCGACCTATGTCGTTTCCTTATCAGCACACTTCACGT |
| Clg1_ORF_3' | GGCAGACTCTAAGTCATTGCATAGCGATTGTACACT |
| Clg1 5'+ InvU4-ASS | GAACAGAATAAATTAGATGTCAAAAAGTTTCGTCAGTCTCGGAGGTAGT AGTGGCGGTAT |
| GBD_pef1_5' | GGCGGATCCGTATGAACTACCAAAGGCTTGAAAAGTTAGGAGAGGGAA CATATGCGCATG |
| Pef1_2_exon | GAGAGGGAACATATGCGCATGTTTATAAGGG |
| Pef1_ORF_3' | GGCGTCGACTATGCGGTTAAAAACCAAGCATGTTGA |
| pREP1_FLAG_S | TATGGACTACAAAGACGATGACGACAAGGGAGGTGCGGCCGCTGTCGACGCTAGCG |
| pREP1_FLAG_AS | GATCCGCTAGCGTCGACAGCGGCCGCACCTCCCTTGTCGTCATCGTCTTTGTAGTCCA |
| Clg1_5'_Not I | GCCGCGGCCGCTATGTCGTTTCCTTATCAGCACACT |
| Clg1_3'_BamH I | GCCGGATCCTCACTAAGTCATTGCATAGCGATT |
| Cek1_5'_Not I | GCCGCGGCCGCTATGAAGCATATAAAAAACGAACGCG |
| Cek1_3'_BamH I | GCCGGATCCTCAAGAGTGCCAAACATCTAACTT |
| Ppk18_5'_Not I | GCCGCGGCCGCTATGGTAATGCAAGAACGCAATTCC |
| Ppk18_3'_Nhe I | GCCGCTAGCTCATTTATTGCAAAGCCGGGCTAT |

**^a^** N = A, T, C or G; W = A or T; S = G or C
