# Supplementary material for: Identification of a Lifespan Extending Mutation in the Schizosaccharomyces pombe Cyclin Gene clg1 + by Direct Selection of Long-Lived Mutants
Source: PLoS One. 2013 Jul 9;8(7):e69084. doi: 10.1371/journal.pone.0069084 (PMC3711543; doi:10.1371/journal.pone.0069084)
Supplement: References S1 — (DOCX) [file pone.0069084.s017.docx]

**Supplemental References**

1. Fabrizio P, Hoon S, Shamalnasab M, Galbani A, Wei M, et al. (2010) Genome-wide screen in Saccharomyces cerevisiae identifies vacuolar protein sorting, autophagy, biosynthetic, and tRNA methylation genes involved in life span regulation. PLoS Genet 6: e1001024

2. Tanaka K, Okayama H (2000) A pcl-like cyclin activates the Res2p-Cdc10p cell cycle "start" transcriptional factor complex in fission yeast. Mol Biol Cell 11: 2845-2862.

3. Martin-Castellanos C, Blanco M, Rozalen AE, Perez-Hidalgo L, Garcia AI, et al. (2005) A large-scale screen in S. pombe identifies seven novel genes required for critical meiotic events. Curr Biol 15: 2056-2062.

4. Mata J, Lyne R, Burns G, Bahler J (2002) The transcriptional program of meiosis and sporulation in fission yeast. Nat Genet 32: 143-147.

5. Saberianfar R, Cunningham-Dunlop S, Karagiannis J Global gene expression analysis of fission yeast mutants impaired in Ser-2 phosphorylation of the RNA pol II carboxy terminal domain. PLoS One 6: e24694.

6. Karagiannis J, Balasubramanian MK (2007) A cyclin-dependent kinase that promotes cytokinesis through modulating phosphorylation of the carboxy terminal domain of the RNA Pol II Rpb1p sub-unit. PLoS One 2: e433.

7. Molz L, Booher R, Young P, Beach D (1989) cdc2 and the regulation of mitosis: six interacting mcs genes. Genetics 122: 773-782.

8. Molz L, Beach D (1993) Characterization of the fission yeast mcs2 cyclin and its associated protein kinase activity. Embo J 12: 1723-1732.

9. Forsburg SL, Nurse P (1991) Identification of a G1-type cyclin puc1+ in the fission yeast Schizosaccharomyces pombe. Nature 351: 245-248.

10. Forsburg SL, Nurse P (1994) Analysis of the Schizosaccharomyces pombe cyclin puc1: evidence for a role in cell cycle exit. J Cell Sci 107 ( Pt 3): 601-613.

11. Samuelsen CO, Baraznenok V, Khorosjutina O, Spahr H, Kieselbach T, et al. (2003) TRAP230/ARC240 and TRAP240/ARC250 Mediator subunits are functionally conserved through evolution. Proc Natl Acad Sci U S A 100: 6422-6427.

12. PomBase [[http://www.pombase.org/spombe/result/SPAC1296.05c]](http://www.pombase.org/spombe/result/SPAC1296.05c%5d).

13. Martin-Castellanos C, Labib K, Moreno S (1996) B-type cyclins regulate G1 progression in fission yeast in opposition to the p25rum1 cdk inhibitor. Embo J 15: 839-849.

14. Bueno A, Richardson H, Reed SI, Russell P (1991) A fission yeast B-type cyclin functioning early in the cell cycle. Cell 66: 149-159.

15. Bueno A, Russell P (1993) Two fission yeast B-type cyclins, cig2 and Cdc13, have different functions in mitosis. Mol Cell Biol 13: 2286-2297.

16. Mondesert O, McGowan CH, Russell P (1996) Cig2, a B-type cyclin, promotes the onset of S in Schizosaccharomyces pombe. Mol Cell Biol 16: 1527-1533.

17. Booher R, Beach D (1988) Involvement of cdc13+ in mitotic control in Schizosaccharomyces pombe: possible interaction of the gene product with microtubules. Embo J 7: 2321-2327.

18. Hagan I, Hayles J, Nurse P (1988) Cloning and sequencing of the cyclin-related cdc13+ gene and a cytological study of its role in fission yeast mitosis. J Cell Sci 91 ( Pt 4): 587-595.

19. Sajiki K, Hatanaka M, Nakamura T, Takeda K, Shimanuki M, et al. (2009) Genetic control of cellular quiescence in S. pombe. J Cell Sci 122: 1418-1429.

20. Guiguen A, Soutourina J, Dewez M, Tafforeau L, Dieu M, et al. (2007) Recruitment of P-TEFb (Cdk9-Pch1) to chromatin by the cap-methyl transferase Pcm1 in fission yeast. Embo J 26: 1552-1559.

21. Pei Y, Schwer B, Shuman S (2003) Interactions between fission yeast Cdk9, its cyclin partner Pch1, and mRNA capping enzyme Pct1 suggest an elongation checkpoint for mRNA quality control. J Biol Chem 278: 7180-7188.

22. Malapeira J, Moldon A, Hidalgo E, Smith GR, Nurse P, et al. (2005) A meiosis-specific cyclin regulated by splicing is required for proper progression through meiosis. Mol Cell Biol 25: 6330-6337.

23. Cameroni E, Hulo N, Roosen J, Winderickx J, De Virgilio C (2004) The novel yeast PAS kinase Rim 15 orchestrates G0-associated antioxidant defense mechanisms. Cell Cycle 3: 462-468.

24. Samejima I, Yanagida M (1994) Identification of cut8+ and cek1+, a novel protein kinase gene, which complement a fission yeast mutation that blocks anaphase. Mol Cell Biol 14: 6361-6371.

25. Vidan S, Mitchell AP (1997) Stimulation of yeast meiotic gene expression by the glucose-repressible protein kinase Rim15p. Mol Cell Biol 17: 2688-2697.
